# Electron Beam Irradiation for Efficient Antibiotic Degradation in Aqueous Solutions

**DOI:** 10.3390/antibiotics14080833

**Published:** 2025-08-15

**Authors:** Anastasia Oprunenko, Ulyana Bliznyuk, Victoria Ipatova, Alexander Nikitchenko, Igor Gloriozov, Arcady Braun, Timofey Bolotnik, Polina Borshchegovskaya, Elena Kozlova, Irina Ananieva, Igor Rodin

**Affiliations:** 1 Department of Chemistry, Lomonosov Moscow State University, GSP-1, 1-3 Leninskiye Gory, 119991 Moscow, Russia; gloriozov@nmr.chem.msu.ru (I.G.); avbraun@yandex.ru (A.B.); timab@tut.by (T.B.); irishan@mail.ru (I.A.); igorrodin@yandex.ru (I.R.); 2 Department of Physics, Lomonosov Moscow State University, GSP-1, 1-2 Leninskiye Gory, 119991 Moscow, Russia; uabliznyuk@gmail.com (U.B.); nikitchenko.ad15@physics.msu.ru (A.N.); alexeevapo@mail.ru (P.B.); 3 Skobeltsyn Institute of Nuclear Physics, Lomonosov Moscow State University, GSP-1, 1-2 Leninskiye Gory, 119991 Moscow, Russia; ipatova.vs15@physics.msu.ru; 4 Department of Medical and Biological Physics, Federal State Autonomous Educational Institution of Higher Education I.M. Sechenov First Moscow State Medical University of the Ministry of Health of the Russian Federation (Sechenovskiy University), 8-2 Trubetskaya str., 119991 Moscow, Russia; waterlake@mail.ru; 5 Lomonosov Institute of Fine Chemical Technologies, MIREA–Russian Technological University, Vernadsky Ave., 78, 119571 Moscow, Russia

**Keywords:** water irradiation, electron beam irradiation (EBI), antibiotics, high-performance liquid chromatography–mass spectrometry, degradation products, DFT calculation

## Abstract

**Background:** Recently, extensive use of antibiotics has increased the amount of antibiotic residues in the natural water environment. **Methods:** This study presents an experimental investigation into the degradation of penicillins, tetracyclines, streptomycin and chloramphenicol in aqueous solutions when exposed to 1 MeV accelerated electrons with doses of 0.1, 1, 3 and 7 kGy using HPLC-HRMS analysis. **Results:** It was found that electron beam irradiation with a dose of 7 kGy ensures 98–99% removal of antibiotics, with the initial concentrations ranging from 15 mg/L to 30 mg/L depending on the class of antibiotic. The mathematical model proposed in the study, which estimates the dose dependencies of the relative concentrations of antibiotics and their degradation products in aqueous solutions, reveals different decomposition rates of antibiotics of different classes due to the different radiosensitivities of antibiotics. It has been found that tetracycline has a considerably higher radiation–chemical yield compared to the other antibiotics when exposed to accelerated electrons. **Conclusions:** Using density functional theory in combination with the mathematical model, we have developed a novel approach to establishing a quantitative irradiation marker of antibiotic degradation as a result of irradiation, which involves finding the degradation product whose formation requires a minimum number of ionization events. Using such an approach, it is possible to establish the extent of antibiotic degradation in water after irradiation with different doses and find the optimal irradiation doses for industrial water treatment.

## 1. Introduction

Recently, extensive use of antibiotics in medicine, agriculture and livestock farming has increased the amount of antibiotic residues in aquatic environments, which has raised serious concerns in the scientific community [1,2]. Recent reports have estimated that antibiotics consumed worldwide in 2018 alone amounted to 40 billion defined daily doses (DDDs), which is a 46% increase since 2000 [3]. Moreover, global antibiotic consumption is projected to be 200% higher in 2030 than in 2015, with the greatest growth in low- and middle-income countries [4]. Most of the antibiotics consumed by humans or animals are not fully metabolized in their bodies and are excreted into the environment through feces and the urinary tract. The increasing overutilization of antibiotics in various sectors is becoming a major contributor to antibiotic pollution in the natural environment since antibiotics linger in the environment as they are not easily biodegradable [5,6,7,8].

As regulators and environmental agencies increasingly recognize the importance and urgency of antibiotic accumulation in the natural environment, developing effective methods to remove antibiotics from water has become a priority. Antibiotic residues are partially eliminated in wastewater treatment plants, but after entering water bodies and wastewater, the remaining metabolites can contribute to the development of drug-resistant bacteria, which pose a serious threat to human health [9,10,11]. Biological processes, which are widely used these days, such as conventional activated sludge wastewater treatment, effectively remove pollutants from wastewater using microorganisms. However, these methods are not suitable for removing pharmaceuticals, pathogens and antibiotic-resistant genes (ARGs), which are discharged into the environment along with secondary effluents [12]. Various treatment methods, including physical and chemical types, have been studied and applied to remove antibiotics from various media to minimize their impact on the environment, including adsorption, coagulation, membrane separation, intense oxidation, etc. [13,14,15]. In recent years, advanced oxidation methods (AOPs) have been increasingly studied and applied to remove antibiotics due to several advantages, such as high reaction rate, high efficiency and productivity [16,17]. UV and photocatalysis, Fenton oxidation, ozone oxidation and peroxide catalytic oxidation, among others, inevitably produce intermediates or byproducts of antibiotics that are more stable and toxic than the parent compounds, so they are limited in terms of degradation efficiency [18].

Compared with traditional processing technologies, electron beam irradiation is more efficient since it obviates the need to use other reagents in the processing and does not cause secondary pollution. Moreover, this highly automated and fast-speed technology allows the irradiation of large volumes of substances while ensuring complete degradation of toxic and persistent organic pollutants [19,20], which makes electron beam irradiation universally applicable in a vast number of areas, ranging from medicine to the food industry. E-beam irradiation can be particularly effective for the treatment of liquid substances due to the dual action of direct ionization by primary accelerated electrons and the indirect action of irradiation of organic pollutants through water radiolysis products [21]. OH• and H• radicals, hydrated electrons $e_{aq}^{-}$, hydrogen H_2_, hydrogen peroxide H_2_O_2_ and hydroxonium ions H_3_O^+^ formed as a result of water radiolysis break chemical bonds of organic pollutants, resulting in their partial or complete decomposition, which is manifested by the release of CO_2_ and H_2_O [8]. Considering the adaptability of electron beam accelerators to different volumes of effluents due to their varying operating modes, electron beam accelerators can be integrated both in small- and large-scale water treatment facilities.

Recent studies on the effect of gamma irradiation on antibiotics of different classes [21,22,23,24,25] attest to the partial or complete decomposition of antibiotics in aqueous solutions. However, there is no clear understanding of the reaction mechanisms and degradation pathways of antibiotics of different classes in water. Considering the current trend to switch from the gamma sources ^60^Co and ^137^Cs to electron accelerators in industrial irradiation facilities, it is important to investigate the efficiency of accelerated electrons for the degradation of antibiotics in aqueous solutions and the mechanisms behind the chemical transformation of antibiotics into antibiotic degradation products.

The object of the study is to explore the impact of e-beam irradiation on a wide range of doses on antibiotics commonly used in medicine, agriculture and livestock farming. In the experiment, tetracycline, doxycycline, benzylpenicillin, amoxicillin, ampicillin, streptomycin and chloramphenicol were diluted in aqueous solutions representing water matrices contaminated with antibiotics. For the identification and quantitative assessment of degradation products of antibiotics, the study used high-performance liquid chromatography combined with high-resolution mass spectrometry (HPLC-HRMS), which is the most promising method for the determination of antibiotics in various matrices and the identification of antibiotic degradation products [26,27]. Another essential goal is to find reliable markers of antibiotic degradation, which would be the basis for an integrated approach to estimating the extent of antibiotic degradation and quantitative assessment of electron beam irradiation efficiency for the treatment of sewage and natural water polluted with antibiotics. Density functional theory (DFT) calculations and mathematical modeling were applied to gain insight into the reaction mechanisms and transformations of antibiotics into the identified degradation products in order to determine the quantitative markers of antibiotic degradation as a result of e-beam irradiation.

## 2. Results and Discussion

### 2.1. The Impact of Accelerated Electrons on Antibiotics in Aqueous Solutions

Immediately after e-beam irradiation, solutions of individual antibiotics were analyzed by HPLC-HRMS to measure the relative concentration of antibiotics and their degradation products. The conditions for recording the mass chromatograms of each antibiotic are presented in Table 1.

**Figure 1:**
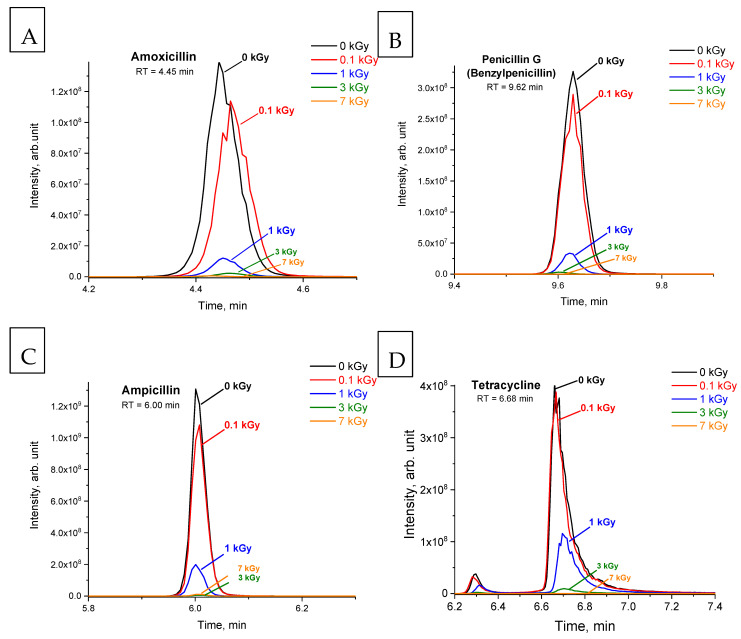

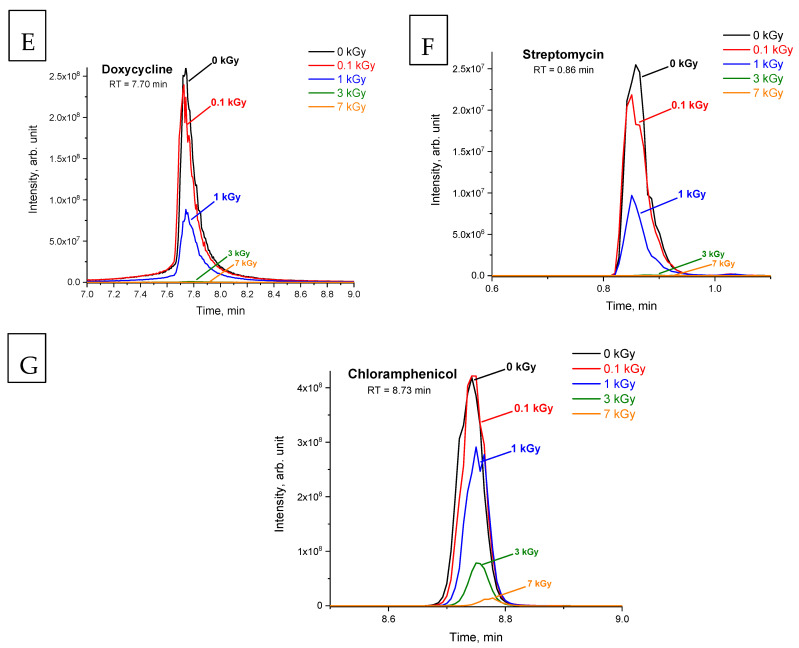
Chromatograms of solutions of amoxicillin (**A**), benzylpenicillin (penicillin G) (**B**), ampicillin (**C**), tetracycline (**D**), doxycycline (**E**), streptomycin (**F**) and chloramphenicol (**G**), non-irradiated (black line) and irradiated with doses of 0.1 (red), 1 (blue), 3 (green) and 7 (orange) kGy.

Figure 1A–G show the chromatograms of non-irradiated (0 kGy) and irradiated (0.1, 1, 3 and 7 kGy) solutions of seven antibiotics and their corresponding retention times. Table 2 represents data on the removal of antibiotics from aqueous solutions under the action of e-beam irradiation. It has been found that irradiation with a dose of 7 kGy can eliminate benzylpenicillin and streptomycin from water, while negligible traces of tetracycline, doxycycline and ampicillin can still be detected. Table 2 also shows that amoxicillin, benzylpenicillin (penicillin G) and ampicillin, belonging to the same class, are the most susceptible to irradiation as they are destroyed, on average, by 20–21% at the minimum dose of 0.1 kGy, while the other antibiotics are decomposed, on average, by not more than 10% at the same dose, with chloramphenicol remaining the most resistant. A two-factor ANOVA analysis was used to assess whether the average value of antibiotic removal varies depending on the irradiation dose and the type of antibiotic. The ANOVA analysis shows that the differences in the average removal values for all antibiotics are significant, with a significance level of *p* = 0.05, which can also be seen in Table 2. Additionally, the Tukey’s range test was performed for each pair of antibiotics, which reveals statistically insignificant differences in the degradation of benzylpenicillin, amoxicillin and ampicillin belonging to the penicillin class of antibiotics.

**Figure 2:**
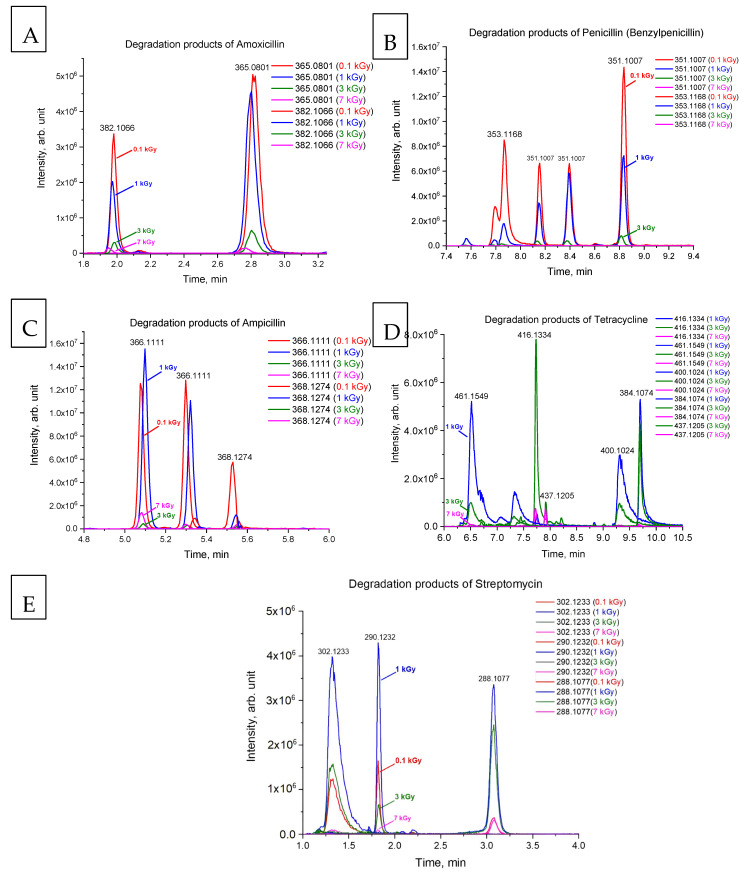
Chromatograms of the degradation products of amoxicillin (**A**), benzylpenicillin (penicillin G) (**B**), ampicillin (**C**), tetracycline (**D**) and streptomycin (**E**).

Figure 2A–E show chromatograms of the degradation products of amoxicillin, benzylpenicillin, ampicillin, tetracycline and streptomycin identified after e-beam irradiation of antibiotic solutions. During the experiment, doxycycline and chloramphenicol degradation products were not detected, which can be explained by the insufficient sensitivity of the suggested method.

Chromatograms 2A and 2B show that the content of degradation products of amoxicillin and benzylpenicillin at the dose of 0.1 kGy reached its maximum. With a further increase in the irradiation dose up to 3 kGy, the degradation products of amoxicillin and benzylpenicillin decreased markedly, and the maximum dose of 7 kGy destroyed the degradation products almost completely, with only a negligible amount traceable. The high radiosensitivity of penicillins can be associated with the presence of a strained beta-lactam ring in their structure [28], which makes them more reactive to water radiolysis species. Other antibiotics demonstrated similar dose behavior involving an increase in the concentration, with a further decrease and with an increase in the irradiation dose. It should be noted that no degradation products of tetracycline were detected at the dose of 0.1 kGy, four degradation products were identified at the dose of 1 kGy, and one degradation product was detected only at 3 kGy. However, the degradation products of other antibiotics, except for doxycycline and chloramphenicol, were found in the aqueous solutions irradiated with the minimum dose of 0.1 kGy. Table 2 shows that chloramphenicol is the most radioresistant to e-beam irradiation since its concentration decreased by under 3% after irradiation with 0.1 kGy, which indicates a low susceptibility to attacks by reactive oxygen species.

Thus, electron beam irradiation with the dose of 7 kGy ensures 98–99% removal of all classes of antibiotics, with the initial concentrations ranging from 15 mg/L to 30 mg/L depending on the antibiotic type. The comparison of the degradation of tetracycline after e-beam irradiation with other common methods of antibiotics removal has shown that while photo-Fenton processing ensures a 94.2% removal of tetracycline from model solutions [29], and a sequencing-batch membrane bioreactor used for swine wastewater treatment ensures 90% removal of tetracycline antibiotics [30], e-beam irradiation with the dose of 7 kGy achieved at least 99.9% elimination in experimental conditions. With a 99% removal rate, e-beam irradiation can be compared with ozonation in terms of its efficiency [31], as ozonation has been reported to achieve 98% removal of oxytetracycline from aquaculture effluents. Although the adsorption methods are simple and inexpensive compared to other methods, they require subsequent treatment of the adsorbent to remove antibiotics from pores, and some amount of antibiotics can enter the watercourse, which makes such methods less sustainable [32,33]. Using photocatalytic degradation technology allows for the decomposition of tetracycline to carbon dioxide and water by over 90% [34]. However, catalysts involved in photocatalytic degradation are specific for a particular antibiotic, which makes it necessary to select the optimal combination of catalysts for each case of effluent water treatment. E-beam irradiation, on the contrary, allows the desired result to be achieved irrespective of the type and combination of antibiotics and without any considerable adjustments while ensuring bacterial purification of water. Considering that e-beam irradiation ensures the removal of antibiotics of all classes and combinations, it can be regarded as a more reliable and scalable water treatment method.

### 2.2. A Mathematical Model Describing the Dependency of the Concentrations of Antibiotics and Degradation Products on Irradiation Dose

**Figure 3:**
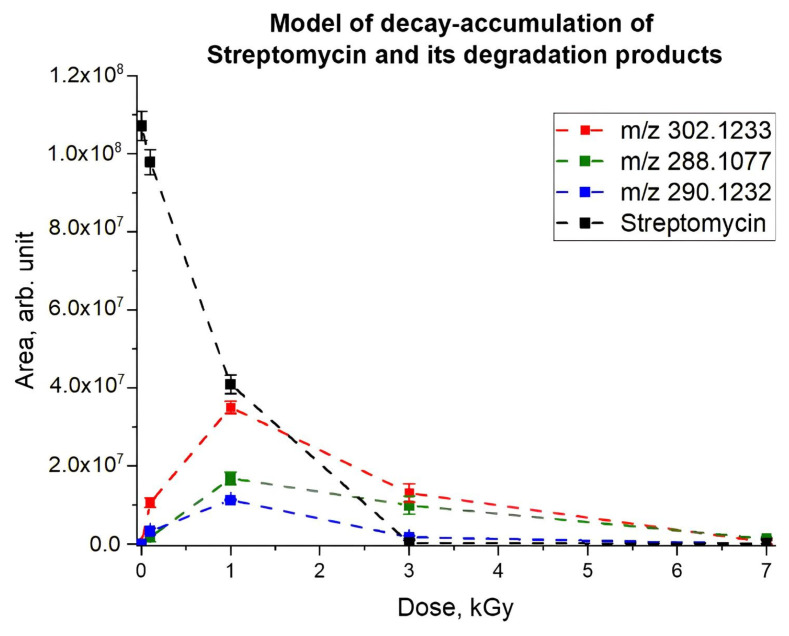
Dependencies of the absolute areas of the chromatographic peaks of streptomycin (black line) and its degradation products (ions with *m*/*z* values of 302.1233, 288.1077 and 290.1232 are shown in red, green and blue lines, respectively) on the radiation dose.

As can be seen from the chromatograms (Figure 2A–E), after irradiation of the antibiotic solutions, the initial concentration of antibiotics decreased to negligible values, while the content of their degradation products increased, reaching its maximum at 0.1–3 kGy depending on the class of antibiotics. Further increase in the irradiation dose to 7 kGy led to nearly complete elimination of antibiotics and their degradation products (Figure 3). The non-linear effect of the irradiation dose on the rate of decomposition of each antibiotic and the rate at which their degradation products are accumulated are described using mathematical modeling.

The exponential decrease in antibiotic concentration with increasing dose can be described by the following differential equation:

(1) $$\left\{\begin{array}{c} \frac{dC_{a}}{dD}=-\alpha C_{a}\\ C_{a}\left(0\right)=C_{0} \end{array}\right.,$$

where α (Gy^−1^) is the decomposition rate of antibiotic molecules per unit of the absorbed dose, C_0_ (rel.un.) is the concentration of the original antibiotic in the non-irradiated solution, and C_a_ (rel.un.) is the concentration of antibiotic in the solution irradiated with the dose D. The solution of the equation is represented as:

(2) $$C_{a}\left(D_{0}\right)=C_{0}e^{-\alpha \;D_{0}}.\;$$

A significant decrease in the concentration of antibiotic degradation products observed, following an initial increase in the higher irradiation dose, is a sign of two competing processes: the accumulation of the degradation product due to the decomposition of the initial antibiotic and the decomposition of the degradation product itself under the action of accelerated electrons.

The differential equation describing the change in the concentration of any degradation product with increasing irradiation dose can be expressed as follows:

(3) $$\frac{{dC}_{p}}{dD}=-\beta C_{p}+{kC}_{a},$$

where β (Gy^−1^) is the decomposition rate of the degradation product, C_p_ (rel.un.) is the concentration of the degradation product in aqueous solution, and k (Gy^−1^) is a coefficient depending on the decomposition rate of initial antibiotic and ratio of initial antibiotic molecules converted into a specific degradation product. When Equation (2) is incorporated into Equation (3), the latter takes the following form:

(4) $$\frac{{dC}_{p}}{dD}=-\beta C_{p}+kC_{0}e^{-\alpha D}.$$

The solution of the inhomogeneous differential Equation (4) is represented as:

(5) $$C_{p}=(\frac{kC_{0}e^{D(-\alpha +\beta )}}{\beta -\alpha }+P)e^{-\beta D},$$

where P (rel. un.) is the integration constant.

Since different degradation products of antibiotics were identified at different irradiation doses, let us determine the dose D_0_ as the threshold dose for the formation of a specific degradation product. This means that when the aqueous solution is irradiated with doses ranging from 0 to D_0_, the concentration of the degradation product C_p_ is equal to 0. Taking into account the initial condition C_p_(D_0_) = 0, we find the constant P:

(6) $$P=\frac{kC_{0}e^{D_{0}(-\alpha +\beta )}}{\alpha -\beta }.$$

Then, the solution of Equation (4) takes the following form:

(7) $$C_{p}=\frac{kC_{0}}{\beta -\alpha }\left(e^{D\left(-\alpha +\beta \right)}-e^{D_{0}\left(-\alpha +\beta \right)}\right)e^{-\beta D}.$$

Solution (8) can be represented using the Heaviside function:

(8) $$C_{p}=H\left(D-D_{0}\right)\frac{kC_{0}}{\beta -\alpha }\left(e^{D\left(-\alpha +\beta \right)}-e^{D_{0}\left(-\alpha +\beta \right)}\right)e^{-\beta D}.$$

Since the degradation products of streptomycin, amoxicillin, ampicillin and benzylpenicillin (Figure 2A–C,E) were identified at the dose of 0.1 kGy, dose D_0_ for these classes of antibiotic is equal to 0, and the solution of Equation (4) is represented as follows:

(9) $$C_{p}=\frac{kC_{0}}{\beta -\alpha }\left(e^{D\left(-\alpha +\beta \right)}-1\right)e^{-\beta D}.$$

**Figure 4:**
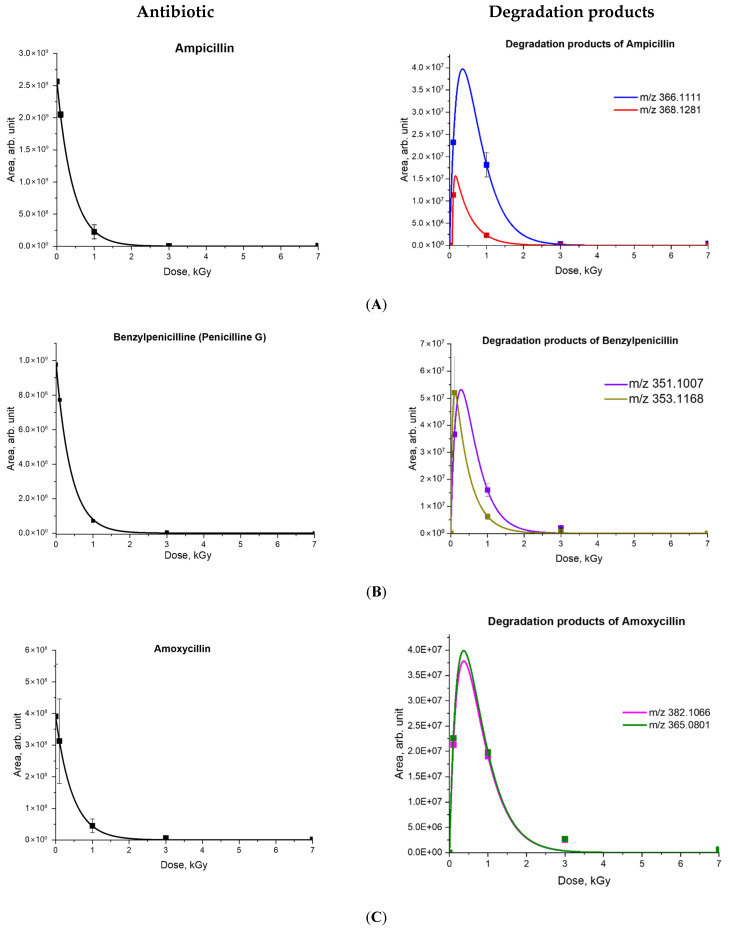

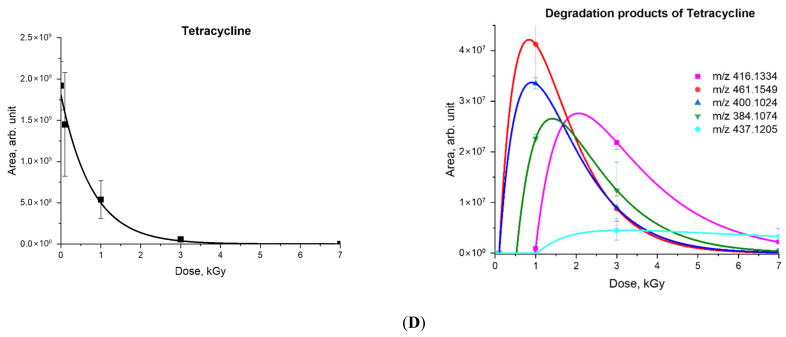
Dependencies of the absolute peak areas of the initial antibiotics and their degradation products on the irradiation dose for ampicillin (**A**), benzylpenicillin (**B**), amoxicillin (**C**) and tetracycline (**D**).

Figure 4 shows the experimental dependencies of the absolute peak areas of the initial antibiotics and their degradation products on the irradiation dose and the dependencies calculated using Formulas (8) and (9). As can be seen, approximation dependencies adequately describe the experimental data, revealing the decomposition of original antibiotics and accumulation and decomposition of degradation products as a result of e-beam irradiation. Table 3 shows the values of the coefficients C_0_, α, β, D_0_ and k, calculated using Equations (1) and (4), for experimental data on the relative concentrations of antibiotics irradiated with accelerated electrons and the detected degradation products. While benzylpenicillin has a higher decomposition rate compared to other antibiotics studied, doxycycline showed the lowest rate of decomposition as a result of irradiation (Table 3). A low margin of error in the approximation coefficients represented in Table 3 attests to the adequacy of the suggested model.

The model suggested above allows the determination of the doses at which not only the initial antibiotics contained in water but also their degradation products are removed from the water. On the other hand, factoring in the concentrations of antibiotic degradation products in the water irradiated with different doses in the mathematical model makes it possible to determine the initial level of the water contamination with antibiotics.

### 2.3. Antibiotic Degradation Pathways in Water Under E-Beam Irradiation Using Tetracycline as an Example

Table 4 shows 15 degradation products (DPs) of tetracycline, amoxicillin, ampicillin, benzylpenicillin and streptomycin identified in aqueous solutions after e-beam irradiation; however, of all of them, tetracycline decomposed into the largest variety of different degradation products as revealed at different irradiation doses. Therefore, further in this article, we focus on possible e-beam irradiation-induced mechanisms of tetracycline decomposition.

Since irradiation of the tetracycline solution had a higher radiation–chemical yield compared to other antibiotics, the dataset of tetracycline degradation products was analyzed on the software package PRIRODA04, using the density functional method (DFT) to make theoretical assumptions regarding the formation of degradation products from the original tetracycline. The DFT method, factoring in thermodynamic calculations of radiation-induced chemical reactions involving antibiotics, provides a molecular-level explanation of the reasons why certain antibiotic degradation products occur. This knowledge is critical for designing an effective water irradiation methodology for eliminating antibiotic residual ecotoxicity.

As can be seen from Figure 1D, the initial tetracycline molecule shows a pronounced chromatographic peak at 6.68 min with the *m*/*z* value of 445.1592 (the TIC chromatogram and ESI mass spectra of tetracycline are shown in Figure S1 in Supplementary Materials). At a dose of 0.1 kGy, the intensity and peak area of tetracycline decreased by 6.2%, and no degradation products were observed. At a dose of 1 kGy, the degradation products with *m*/*z* values of 461.1549 (DP-TC-460), 400.1024 (DP-TC-399), 384.1074 (DP-TC-383) and 416.1334 (DP-TC-415) appeared (Figure 4D). When exposed to 3 kGy, the degradation product with an *m*/*z* value of 437.1205 (DP-TC-436) peaked in the mass chromatogram.

**Figure 5:**
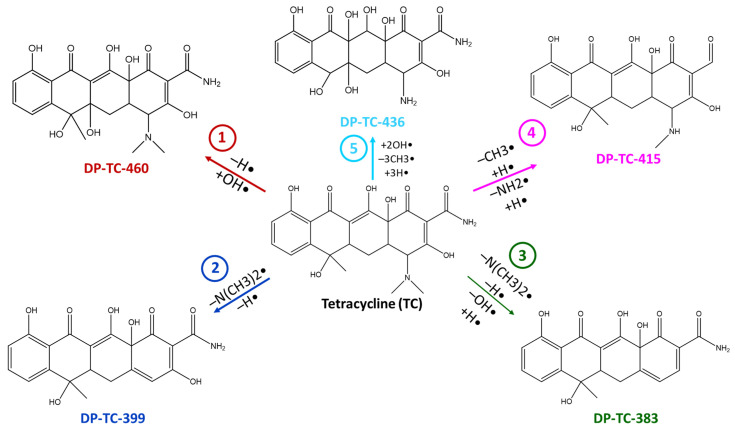
The most probable pathways of tetracycline degradation under the action of accelerated electrons.

The degradation products identified in water occur as a result of different radiation-induced reactions, and the most probable degradation pathways for tetracycline are shown in Figure 5. The analysis of the structures and the type of degradation products suggests that the main degradation pathways of tetracycline are demethylation, deaminomethylation and dehydroxylation, as well as hydroxylation, occurring without disturbing the ring structure of the original molecule.

**Figure 6:**
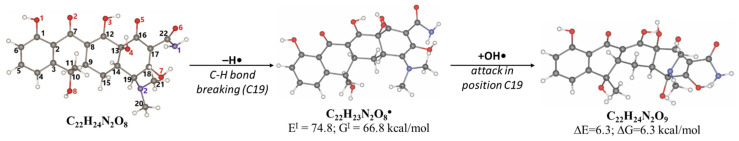
The optimized structures of tetracycline and its degradation product with an *m*/*z* value of 461.1549 (DP-TC-460) and energies of the formation of the intermediate and final product. Carbon atoms are grey, hydrogen atoms are white, oxygen atoms are red, and nitrogen atoms are violet.

As can be seen from Figure 4D, the concentration of the product DP-TC-460 in the water irradiated with 1 kGy was higher than the concentrations of the other degradation products DP-TC-399, DP-TC-383 and DP-TC-415 identified at this dose. It can be assumed that the product DP-TC-460 is a hydroxylated product of tetracycline with a substituted hydroxyl radical OH• instead of the H atom (Figure 6). Hydroxyl radical OH•, formed as a result of water radiolysis, attacks the tetracycline molecule, and the molecular structure of tetracycline can provide at least seven theoretically possible positions of the OH group (seven isomers). The criterion for the formation of the products can be the energy of breaking the C-H bond at various carbon atoms: C4, C5, C6, C9, C14, C15 and C19. The energy of C-H bond-breaking ∆E^I^ and Gibbs energy ∆G^I^, revealing the nature of the chemical reaction, are calculated using the following equations:

Tetracycline (TC) → Radical (Cn)● + H●(10)

∆E^I^ = E(Radical(Cn) ●) + E(H●) − E(TC) (11)

∆G^I^ = ∆E^I^ − [G(Radical(Cn) ●) + G(H●)] − G(TC)], (12)

where ∆E^I^, kcal/mol is the total energy of compounds, and ∆G^I^, kcal/mol is Gibbs energy.

According to Table 5, the bond dissociation energies at carbon atoms C4, C5, C6, C9, C14, C15 and C19 show that the hydrogen atom at position 19 is prone to dissociation due to a low bond energy, which leads to the formation of DP-TC-460 upon attack by the hydroxyl radical OH• at position 19. The energies of the remaining isomers of the hydroxylated product DP-TC-460 are given in Table S2 in Supplementary Materials. The energy of the isomer with the hydroxyl position at C19 is 6.3 kcal/mol higher compared to that with the hydroxyl position at C5, which suggests that this configuration does not yield any energy gain. It can be assumed that the breaking of the C-H bond plays a major role in the formation of the primary radical (Cn)●, since the dissociation energy of hydrogen from C19 is 39 kcal/mol lower than for C5. Thus, the DFT method shows that DP-TC-460 is formed due to two consecutive events: direct ionization manifested in the breaking of the C-H bond and the interaction of OH• with the primary radical (Cn)●.

**Figure 7:**
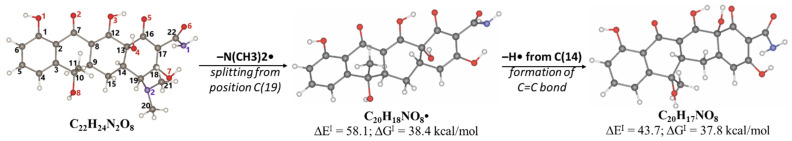
The dissociation of the dimethylamino group from position C(19) to form DP-TC-399 with an *m*/*z* value of 400.1024. Carbon atoms are grey, hydrogen atoms are white, oxygen atoms are red, and nitrogen atoms are violet.

A slightly lower concentration of the degradation product DP-TC-399 with *m*/*z* 400.1024 compared to DP-TC-460 was identified in water irradiated with 1 kGy (Figure 7). It can be assumed that the tetracycline molecule transforms into the degradation product DP-TC-399 by losing C_2_H_8_N, which results in the loss of the dimethylamine group. DFT calculations show that the dimethylamine group breaks off from the C19 position, and the dissociation requires the energy of 38.4 kcal/mol. The second step in the formation of DP-TC-399 is the dissociation of the hydrogen radical H•, with 37.8 kcal/mol expended in the process. Thus, the decomposition of the tetracycline molecule with the formation of the degradation product DP-TC-399 involves two direct ionizations, resulting in the breaking of the bond C19-N(CH_3_)_2_ and dissociation of the hydrogen radical H•.

**Figure 8:**
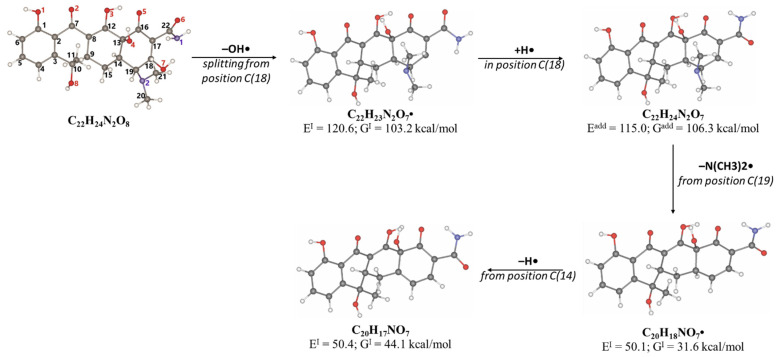
Formation of product DP-TC-383 with *m*/*z* value of 384.1074. Carbon atoms are grey, hydrogen atoms are white, oxygen atoms are red, and nitrogen atoms are violet.

The degradation product DP-TC-383 is formed from DP-TC-399 by the elimination of the hydroxyl group, presumably at C18, and the subsequent addition of the hydrogen radical H• (Figure 8). Then, the dimethylamino group is eliminated from position C19 and the hydrogen radical H• from position C14. The total energy gain of this process is 72.8 kcal/mol (see Figure S37 in Supplementary Materials). It should be noted that the degradation products DP-TC-460, DP-TC-399 and DP-TC-383 were found in tetracycline solution irradiated with ultraviolet, which attests to the consistency of our findings [35].

**Figure 9:**
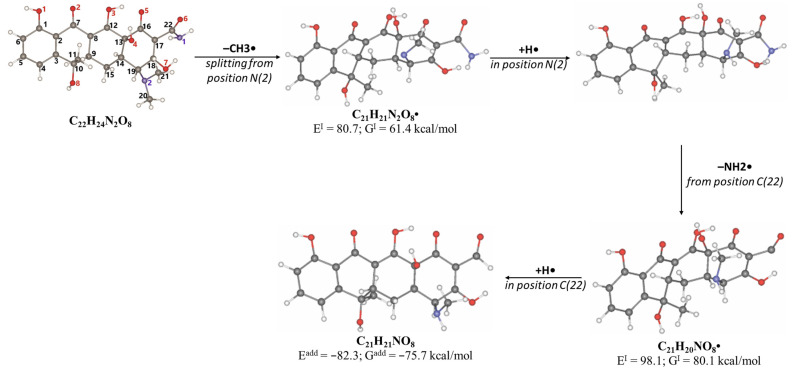
The formation of degradation product DP-TC-415 with an *m*/*z* value of 416.1334. Carbon atoms are grey, hydrogen atoms are white, oxygen atoms are red, and nitrogen atoms are violet.

The product DP-TC-415 is obtained by the successive elimination of one methyl radical from the nitrogen atom in the dimethylamino group and the amino group from C22 (Figure 9). This process occurs with a decrease in energy by 22.2 kcal/mol compared to the original tetracycline molecule (see Figure S38 in Supplementary Materials). Thus, the decomposition of the tetracycline molecule with the formation of the degradation product DP-TC-415 involves three direct ionization events and indirect ionization by hydrogen radical H•. This means that the degradation product DP-TC-415 requires a higher irradiation dose of 3 kGy to form from the original tetracycline molecule as opposed to the aforementioned degradation products, which require 1 kGy to be easily detectable in water.

**Figure 10:**
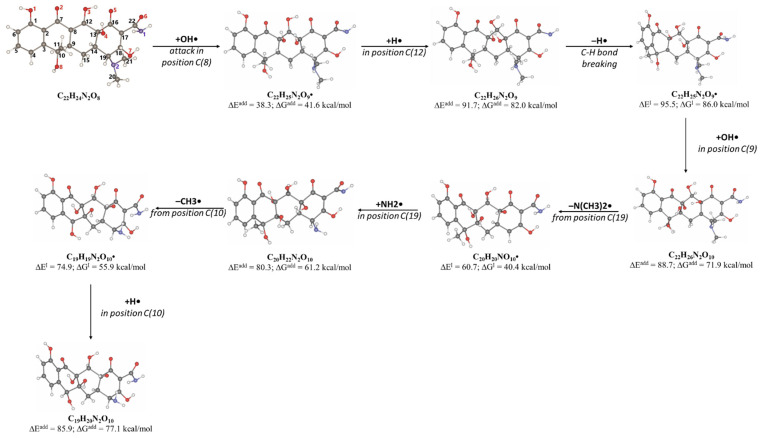
The formation of degradation product DP-TC-436 with an *m*/*z* value of 437.1205. Carbon atoms are grey, hydrogen atoms are white, oxygen atoms are red, and nitrogen atoms are violet.

As can be seen from Figure 4D, the concentration of the degradation product DP-TC-436 is considerably lower compared to other products, and it was found at doses above 3 kGy. Thus, it can be assumed that the formation of DP-TC-436 is a complex process requiring a greater number of ionization events. DFT calculations confirm that DP-TC-436 can be formed as a result of the addition of two hydroxyl radicals OH• at positions C8 and C9, followed by the elimination of the dimethylamino group N(CH_3_)_2_• from C19 and the addition of the amino radical NH_2_• at C19, with a further elimination of the methyl radical CH_3_• from C10 (Figure 10). This process occurs with a decrease in energy by 68.3 kcal/mol compared to the initial tetracycline molecule (see Figure S39 in Supplementary Materials).

Considering the complexity of possible radiation-induced mechanisms of antibiotic degradation in aqueous solutions calculated using the DFT model, resulting in the formation of degradation products, it can be concluded that the degradation products occurring at low doses are easily formed since they require fewer ionization events involving direct ionization by primary electrons and ionization through water radiolysis species compared to other degradation products. Considering that the mathematical model describing the dose-dependencies of the concentrations of antibiotic and degradation products yields more accurate dependencies when it processes the data on the degradation products with shorter formation pathways, such degradation products can be effectively used as markers of the degradation of initial antibiotics. Knowing the concentration of antibiotic degradation markers, the concentration of the initial antibiotic in a water source can be reconstructed to estimate the extent of contamination of the natural environment with antibiotics.

## 3. Materials and Methods

### 3.1. Research Stages

**Figure 11:**
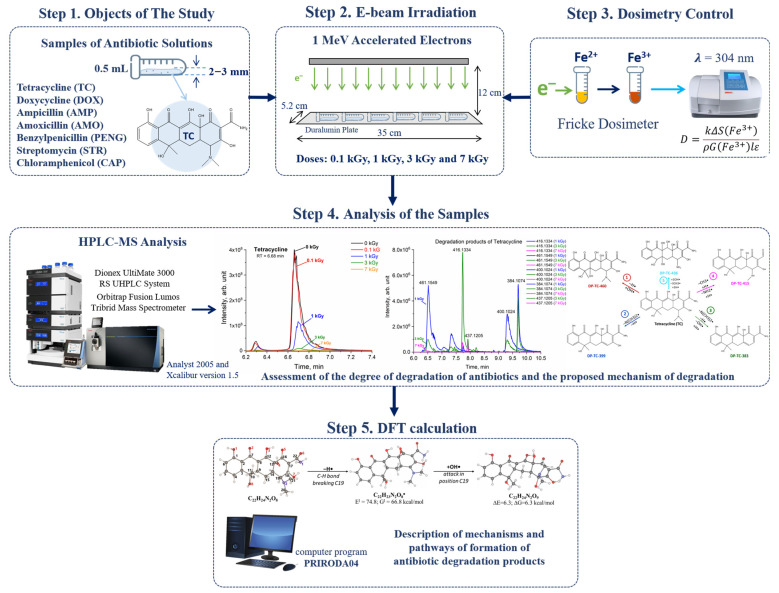
Research stages.

To study the impact of accelerated electrons with different doses on the degradation of antibiotics, aqueous solutions of antibiotics (tetracycline (TC), doxycycline (DOX), ampicillin (AMP), amoxicillin (AMO), benzylpenicillin (PENG), streptomycin (STR) and chloramphenicol (CAP)) were prepared for further irradiation and HPLC-HRMS analysis. The methodology consists of four main steps, including sample preparation, irradiation, dosimetry control and HPLC-HRMS analysis of irradiated and non-irradiated samples (Figure 11). Solutions of each class of antibiotics were distributed into six 0.5 mL Eppendorf tubes, with three iterations for each irradiation dose (Step 1), and then irradiated with doses of 0.1, 1, 3 and 7 kGy (Step 2). The total number of samples was 210, with 30 for each class of antibiotics. Fricke dosimetry was used to estimate the dose absorbed by the samples during irradiation. The uniformity of absorbed dose distribution was calculated using Geant 4 computer simulation (Step 3). After irradiation, the samples were analyzed to study the transformation of the original antibiotics and obtain the structure of their degradation products (Step 4). Density functional method (DFT) calculations were applied to understand the degradation mechanisms of the antibiotics (Step 5).

### 3.2. Reagents, Equipment, Reference Materials and Sample Preparation for Analysis

Reagents

The following reagents were used for the experiment: 98% formic acid (33015–500 ML, ACS reagent puriss. p.a., Sigma-Aldrich, St. Louis, MO, USA); acetonitrile (AC03292500, HPLC-grade, Scharlau, Barcelona, Spain); methanol (EVA-MEM-2.5, for HPLC, Eva Science, Saint Petersburg, Russia); deionized water purified in a Milli-Q system (Millipore, Temecula, CA, USA). Automatic pipettes (5–50 μL, 10–100 μL, 20–200 μL and 100–1000 μL), with a limit of measurement error of no more than ± 5% (Labmate, Chicago, IL, USA), were used to obtain an accurate aliquot. Weighing of accurate reagent suspensions was carried out on the analytical scales ‘Vibra’ with an accuracy of 0.0001 g (Japan).

Equipment

To analyze the degradation of antibiotics in aqueous solutions and quantify the antibiotic content as a function of exposure dose, we used an Ultimate 3000 RSLC liquid chromatograph (Dionex, Germering, Germany) with an automatic sample introduction system and an Orbitrap Fusion Lumos high-resolution mass spectrometer (Thermo Fisher Scientific, Waltham, MA, USA) containing an electrospray ionization source. Chromatographic separation was performed on an Acclaim RS LC HPLC column (150 mm × 2.1 mm, sorbent grain diameter 2.2 μm) manufactured by Dionex (Sunnyvale, CA, USA). A Security Guard C18 precolumn (Phenomenex, Torrance, CA, USA) was installed in the system to extend the lifetime of the chromatographic column. Chromatograms were recorded using Analyst 2005 (AB Sciex MSD, Concord, ON, Canada) and Xcalibur version 1.5 software packages. Also, for preliminary experiments to study the separation of antibiotics and optimize their detection using a mass spectrometer, an HPLC–MS system consisting of an Expec L-Chrom MS HPLC system (Expec, Hangzhou, China) was used.

Sample Preparation

The objects of the study were aqueous solutions of standard samples of seven antibiotics, which are widely used by humans in medical practice: tetracycline (TC)—tetracycline hydrochloride, T3383-25G, Sigma-Aldrich; doxycycline (DOX)—doxycycline hyclate D9891, Sigma; amoxicillin (AMO)—amoxicillin A8523, Sigma-Aldrich; ampicillin (AMP)—ampicillin sodium salt A9518-5G, Sigma-Aldrich; benzylpenicillin (PENG)—penicillin G sodium salt 13752, Sigma; streptomycin (STR)—streptomycin sulfate salt, S6501-5G, Sigma-Aldrich; and chloramphenicol (CAP)—chloramphenicol, C0378, Sigma-Aldrich. The purity of the standards used was above 99%. The physical and chemical properties and structures of the antibiotics are summarized in Table S1 in Supplementary Materials. Stock solutions of antibiotics with concentrations of 850–1000 mg/L were prepared by dissolving suspensions of each antibiotic in an exact volume of solvent, water or methanol, depending on the solubility of the antibiotics.

The concentrations of antibiotics in aqueous solutions prepared for irradiation were TC—21.3 mg/L, DOX—21 mg/L, PENG—18.2 mg/L, AMP—18.3 mg/L, AMO—19.8 mg/L, CAP—15.2 mg/L and STR—29.3 mg/L. All solutions were placed in 2 mL Eppendorf-type plastic microcentrifuge tubes, with 0.5 mL of solution in each Eppendorf tube (OAO RZP, Rybinsk, Russia) for subsequent irradiation. For each antibiotic, 30 Eppendorf tubes were prepared, with three iterations for each radiation dose.

### 3.3. E-Beam Irradiation of Aqueous Solutions

The aqueous solutions containing antibiotics were irradiated with accelerated electrons generated by a continuous electron accelerator UELR-1-25-T001 (Skobeltsyn Research Institute of Nuclear Physics, Moscow State University, Moscow, Russia) with a maximum beam energy of 1 MeV (Figure 2A). The beam current varied from 360 to 2633.3 nA to reduce the exposure time when the samples were irradiated with high doses. For each irradiation session, 6 Eppendorf tubes containing antibiotic solutions were placed on a duralumin plate according to the irradiation method described in [36]. During each irradiation session, to control the dose absorbed by the samples, we measured the charge absorbed by the duralumin plate. The margin of error in the charge absorbed by the plate, measured using ADC (LLC ‘Production Association OVEN’, Moscow, Russia), did not exceed 2%. To ensure uniform dose distribution over the entire volume of the samples due to the low penetration depth of 1 MeV electrons, the height of the solution was no more than 2 mm. Based on Geant 4 computer simulation [36], it was found that the dose uniformity in aqueous solutions when irradiated with 1 MeV electrons was 0.6. A wide dose range from 0.1 kGy to 7 kGy was selected in order to cover the dose range commonly used in water and food irradiation. The ambient temperature during each irradiation session was 20 °C.

### 3.4. Dosimetry Control

A ferrous sulfate Fricke dosimeter was used to measure the doses absorbed by the samples. A total of 0.5 mL of FeSO_4_ solution was placed in 2 mL Eppendorf tubes similar to the experimental ones and irradiated under the same conditions as the antibiotic solutions. The exposure time of the solution was recorded during each session. As a result of the radiolysis of water under the action of free radicals reacting with FeSO_4_ solution, Fe^2+^ ions are oxidized to Fe^3+^ ions, which leads to a change in the optical density of the solution. The concentration of Fe^3+^ ions was estimated by comparing the optical densities of irradiated and non-irradiated samples using a spectrophotometer UV-3000 (TM Ecoview, Moscow, Russia) at a wavelength of 304 nm [37].

Based on the change in optical density, which depends on the transition of Fe^2+^ to Fe^3+^ at irradiation with different doses, the following formula was used to find the doses absorbed by the solutions:

(13) $$D=\frac{k\Delta S({Fe}^{3+})}{\rho G({Fe}^{3+})l\varepsilon },$$

where k = 9.65 × 10^6^ is the dimensionless coefficient, ΔS is the optical density of the Fricke solution, ρ = 1.024 g/cm^3^ is the density of the solution, G(Fe^3+^) = 15. 6 ion/100 eV is the radiation–chemical yield when exposed to accelerated electrons with the energy up to 10 MeV, l = 1 cm is the optical path, and ε = 2160 l/(mol•cm) is the extinction coefficient of Fe^3+^ ions [38].

Table 6 contains the exposure time, beam current, the charge absorbed by the duralumin plate and the dose for twelve irradiation sessions. The margin of error in each irradiation parameter was no more than 5%. The samples were irradiated with doses of 0.1 kGy, 1 kGy, 3 kGy and 7 kGy.

### 3.5. HPLC-MS Analysis

Immediately after irradiation, the antibiotic solutions were subjected to HPLC-MS analysis. Two 0.5 mL antibiotic solutions from each Eppendorf tube irradiated with the same dose were placed in 1.5 mL chromatographic vials. The electrospray ionization source was used in the mode of registration of positively charged or negatively charged ions. The resolution of the mass analyzer was not less than 30,000 rel.un., and the error in determining *m*/*z* values did not exceed 5 ppm. Chromatographic separation was carried out in the gradient elution mode. The mobile phase A was 0.1% formic acid, and the mobile phase B was acetonitrile. All the conditions of HPLC-MS analysis are summarized in Table A1 in Appendix A.

### 3.6. Density Functional Theory (DFT) Calculation

To calculate the most probable transformation pathways of antibiotics to the identified degradation products, DFT calculations were used.

The geometries of molecules were fully optimized by means of density functional theory (DFT) calculations. We used first-principles PBE functionals [39]. The full electron basis set L1 was used, where L1 stands for double set size. The numbers of contracted and primitive functions used in L1 are, respectively, {2,1}/{6,2} for H and {3,2,1}/{10,7,3} for C, N and O [40]. Stationary points on the potential energy surface (PES) were identified by analyzing Hessians. The thermodynamic functions (Gibbs energies, G) at 298.15 K were calculated using an approximation of a restricted rotator and harmonic oscillator. All calculations were performed using a personal computer with the use of the PRIRODA04 program written by Laikov [41].

## 4. Conclusions

The study focuses on determining the doses of electron beam irradiation at which seven antibiotics commonly used in medicine—ampicillin, amoxicillin, benzylpenicillin, streptomycin, tetracycline, doxycycline and chloramphenicol—are removed from water. It has been proven that 1 MeV electron beam irradiation with a dose of 7 kGy ensures 98–99% removal of antibiotics, with the initial concentrations ranging from 15 mg/L to 30 mg/L depending on the antibiotic type. It should be noted that the antibiotics selected for the study have different radiosensitivities. While in the case of ampicillin, amoxicillin and benzylpenicillin, their degradation products were completely eliminated from the water after irradiation with 7 kGy, the degradation products of streptomycin and tetracycline were still present in negligible amounts in the water after irradiation with the same dose. A mathematical model used in the study, describing the monotonous decline of antibiotics and non-monotonous behavior of antibiotic degradation products depending on the irradiation dose, determined that the decomposition rate of antibiotics of the penicillin class is, on average, two times higher than that of streptomycin and tetracycline. In contrast, chloramphenicol with an initial concentration of 15.15 mg/L had the highest resistance to e-beam irradiation, and even the highest dose of 7 kGy could not completely eliminate it from the water, and at the same time, its decomposition rate was the lowest.

It has been found that different degradation products of antibiotics are detected at different irradiation doses. Density functional theory calculations have proven that the presence of the threshold dose at which specific degradation products are found is determined by the number of ionization events triggering the chemical transformation of antibiotic molecules. The degradation products requiring only one or two direct or indirect ionization events can serve as potential markers of antibiotic degradation since the mathematical model proposed in the study allows the dose dependencies of the initial antibiotics to be reconstructed with high accuracy.

Future studies will validate the application of electron beam irradiation for the treatment of wastewater and foods, assess the toxicological safety of degradation products, and optimize electron beam irradiation efficiency. The radiation-induced mechanisms of antibiotic degradation, confirmed by DFT calculations, open the way to more effective methods of radiation–chemical purification of water that may contain antibiotics and other drugs. Our further research will aim at gaining a deeper understanding of the combined action of ozonation and electron beam irradiation to enhance the efficiency of the removal of antibiotics and degradation products when treating water sources or biological substances. Further, we intend to study the impact of water quality parameters on antibiotic degradation as a result of irradiation to understand how varying water parameters can affect the outcome of irradiation efficiency. Such studies will accelerate the practical deployment of electron beam irradiation to ensure water and food safety in response to the challenges faced by the natural environment due to the overuse and incorrect disposal of antibiotics.

## Figures and Tables

**Table 1:** Chromatography–mass spectrometric characteristics of antibiotic identification in aqueous solutions (standard deviation of peak areas is not more than 20%, n = 3).

Antibiotic	*m*/*z*	Polarity	Retention Time (RT), min	Peak Area (0 kGy), arb. unit.
Amoxicillin (Figure 1A)	366.1110	[M+H]^+^	4.45	3.7 × 10^8^
Penicillin G (Figure 1B)	335.1056	[M+H]^+^	9.62	9.7 × 10^8^
Ampicillin (Figure 1C)	350.1163	[M+H]^+^	6.00	2.5 × 10^9^
Tetracycline (Figure 1D)	445.1592	[M+H]^+^	6.68	1.9 × 10^9^
Doxycycline (Figure 1E)	445.1594	[M+H]^+^	7.70	4.5 × 10^9^
Streptomycin (Figure 1F)	582.2725	[M+H]^+^	0.86	7.4 × 10^7^
Chloramphenicol (Figure 1G)	321.0050	[M+H]^−^	8.73	2.4 × 10^9^

**Table 2:** Removal of antibiotics from aqueous solutions (in %) depending on irradiation dose (n = 3, *p* = 0.95). ^1^ Not detected.

Antibiotic	Removal, %				
	0 kGy	0.1 kGy	1 kGy	3 kGy	7 kGy
Amoxicillin (Figure 1A)	0	21.5 ± 3.1	88.4 ± 8.6	97.8 ± 0.8	98.9 ± 0.4
Penicillin G (Figure 1B)	0	20.8 ± 4.7	92.6 ± 3.3	99.3 ± 0.6	100 ^1^
Ampicillin (Figure 1C)	0	20.1 ± 9.9	91.2 ± 18.4	99.4 ± 1.9	99.6 ± 1.0
Tetracycline (Figure 1D)	0	6.2 ± 2.4	65.3 ± 5.5	97.9 ± 3.3	99.9 ± 0.1
Doxycycline (Figure 1E)	0	8.6 ± 1.8	65.2 ± 14.8	99.4 ± 0.8	99.9 ± 0.1
Streptomycin (Figure 1F)	0	10.1 ± 2.5	61.6 ± 14.4	99.8 ± 0.3	100 ^1^
Chloramphenicol (Figure 1G)	0	2.4 ± 0.5	35.1 ± 8.1	82.8 ± 3.8	98.8 ± 3.4

**Table 3:** Approximation coefficients describing the transformations of antibiotics and their degradation products.

Antibiotic	C_0_	α	R_corr_	Degradation Products	β	D_0_	k
Tetracycline	100	1.28 ± 0.16	0.99	461.1549	0.65 ± 0.08	0.98 ± 0.12	0.14 ± 0.02
				400.1024	1.44 ± 0.18	0.09 ± 0.01	0.10 ± 0.01
				384.1074	1.20 ± 0.15	0.09 ± 0.01	0.07 ± 0.01
				416.1334	0.98 ± 0.12	0.53 ± 0.07	0.09 ± 0.01
				437.1205	0.10 ± 0.01	1.04 ± 0.13	0.015 ± 0.002
Ampicillin	100	2.38 ± 0.24	0.98	366.1111	3.38 ± 0.34	2.33 ± 0.24	0.12 ± 0.01
				368.1274	32.12 ± 3.25	0.07 ± 0.01	0.28 ± 0.03
Amoxicillin	100	2.18 ± 0.84	0.99	365.0801	3.29 ± 1.27	6.01 ± 2.32	0.72 ± 0.26
				382.1066	3.34 ± 1.30	3.08 ± 1.19	0.76 ± 0.29
Benzylpenicillin	100	2.51 ± 0.12	0.99	351.1007	4.97 ± 0.23	3.86 ± 0.18	0.54 ± 0.02
				353.1168	22.77 ± 1.05	0.0022 ± 0.0001	1.60 ± 0.07
Streptomycin	100	1.02 ± 0.50	0.99	302.1233	1.12 ± 0.56	2.39 ± 1.19	0.96 ± 0.48
				290.1232	3.28 ± 1.65	0.05 ± 0.03	0.74 + 0.37
				288.1077	0.68 ± 0.34	0.05 ± 0.03	0.38 ± 0.19
Doxycycline	100	0.56 ± 0.04	0.89	Not detected			
Chloramphenicol	100	1.49 ± 0.08	0.98	Not detected			

**Table 4:** Degradation products of antibiotics identified in antibiotic aqueous solutions after e-beam irradiation.

Antibiotic	Name and Molecular Formula [M+H]^+^	Retention Time, min	Dose, kGy	Accurate Mass [M+H]^+^	Mass Accuracy, ppm	Structure
Tetracycline	DP-TC-460 C_22_H_25_N_2_O_9_	6.51 и 7.31	1	461.1549	−1.31	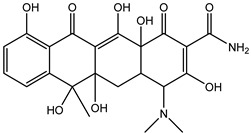
	DP-TC-399 C_20_H_18_NO_8_	9.33	1	400.1024	−0.77	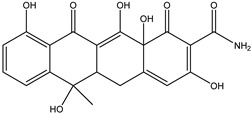
	DP-TC-383 C_20_H_18_NO_7_	9.68	1	384.1074	−0.37	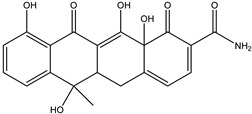
	DP-TC-415 C_21_H_22_NO_8_	7.74	1	416.1334	−1.44	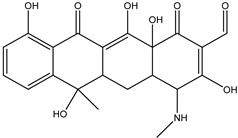
	DP-TC-436 C_19_H_21_N_2_O_10_	7.92	3	437.1205	0.31	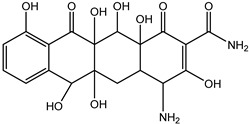
Amoxicillin	DP-AMO-364 C_16_H_17_N_2_SO_6_	2.81	0.1	365.0801	0.07	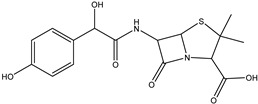
	DP-AMO-381 C_16_H_20_N_3_SO_6_	1.97	0.1	382.1066	−0.33	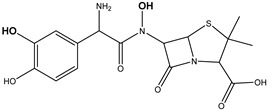
Ampicillin	DP-AMP-365 C_16_H_20_N_3_SO_5_	5.12	0.1	366.1111	−0.37	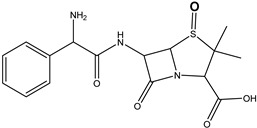
	DP-AMP-367 C_16_H_22_N_3_SO_5_	5.60	0.1	368.1274	0.82	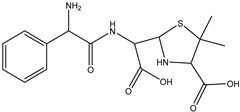
Benzylpenicillin	DP-PENG-350 C_16_H_19_N_2_SO_5_	8.16; 8.40; 8.83	0.1	351.1007	−0.34	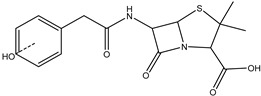
	DP-PENG-352 C_16_H_21_N_2_SO_5_	7.83	0.1	353.1168	0.54	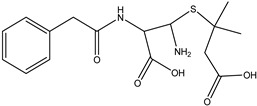
	DP-PENG-308 C_15_H_21_N_2_SO_3_	7.83	0.1	309.1276	−0.2	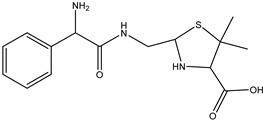
Streptomycin	DP-STR-301 C_13_H_20_O_7_N	1.31	0.1	302.1233	−0.56	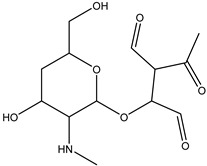
	DP-STR-289 C_12_H_20_O_7_N	1.82	0.1	290.1232	−0.79	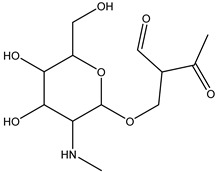
	DP-STR-287 C_12_H_18_O_7_N	3.07	0.1	288.1077	−0.18	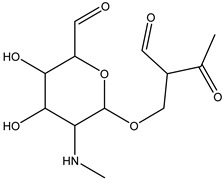

**Table 5:** Total energy E^I^ and Gibbs energy *G^I^* for tetracycline molecules in different positions of carbon atoms (Figure 6).

	C_4_•	C_5_•	C_6_•	C_9_•	C_15_•	C_14_•	C_19_•
E^I^, kcal/mol	113.5	115.2	115.5	77.5	99.7	94.7	74.8
G^I^, kcal/mol	105.4	105.8	106.6	69.4	90.9	85.2	66.8

**Table 6:** Irradiation parameters.

Session	Irradiation Time (avg.), s	Charge (avg.), nC	Dose (avg.), Gy
1–3	32.3	5375	103.4
4–6	122.3	52,356.7	1007
7–9	73.7	157,733.3	3033.3
10–12	150	365,666.7	7032.1

## Data Availability

Data are available within the article, Supplementary Materials and upon request from the corresponding authors.

## Supplementary Materials

The following supporting information can be downloaded at: https://www.mdpi.com/article/10.3390/antibiotics14080833/s1. Figure S1: TIC chromatogram of Tetracycline, retention time(RT) 6.68 min; Figure S2: ESI mass spectra of Tetracycline in the positive ion mode; Figure S3: MS2 spectra of Tetracycline molecular ion m/z 445.1592; Figure S4: MS2 spectra of DP-TC-460 with m/z 461.1549; RT = 6.51 min; detected at a dose of 1 kGy; Figure S5: MS2 spectra of DP-TC-399 with m/z 400.1024; RT = 9.33 min; detected at a dose of 1 kGy; Figure S6: MS2 spectra of DP-TC-383 with m/z 384.1074; RT = 9.68 min; detected at a dose of 1 kGy; Figure S7: MS2 spectra of DP-TC-415 with m/z 416.1334; RT = 7.74 min; detected at a dose of 1 kGy; Figure S8: MS2 spectra of DP-TC-436 with m/z 437.1205; RT = 7.92 min; detected at a dose of 3 kGy; Figure S9: TIC chromatogram of Amoxicillin, retention time 4.45 min; Figure S10: ESI mass spectra of Amoxicillin in the positive ion mode; Figure S11: MS2 spectra of Amoxicillin molecular ion m/z 366.1110; Figure S12: MS2 spectra of DP-AMO-364 with m/z 365.0801; RT = 2.81 min; detected at a dose of 0.1 kGy; Figure S13: MS2 spectra of DP-AMO-381 with m/z 382.1066; RT = 1.97 min; detected at a dose of 0.1 kGy; Figure S14: TIC chromatogram of Ampicillin, retention time 6.00 min; Figure S15: ESI mass spectra of Ampicillin in the positive ion mode; Figure S16: MS2 spectra of Ampicillin molecular ion m/z 350.1163; Figure S17: MS2 spectra of DP-AMP-365 with m/z 366.1111; RT = 5.12 min; detected at a dose of 0.1 kGy; Figure S18: MS2 spectra of DP-AMP-367 with m/z 368.1274; RT = 5.60 min; detected at a dose of 0.1 kGy; Figure S19: TIC chromatogram of Benzylpenicillin, retention time 9.62 min; Figure S20: ESI mass spectra of Benzylpenicillin in the positive ion mode; Figure S21: MS2 spectra of Benzylpenicillin molecular ion m/z 335.1056; Figure S22: MS2 spectra of DP-PENG-350 with m/z 351.1007; RT = 8.16; 8.40; 8.83 min; detected at a dose of 0.1 kGy; Figure S23: MS2 spectra of DP-PENG-352 with m/z 353.1168; RT = 7.83 min; detected at a dose of 0.1 kGy; Figure S24: MS2 spectra of DP-PENG-308 with m/z 309.1276; RT = 7.83 min; detected at a dose of 0.1 kGy; Figure S25: TIC chromatogram of Streptomycin, retention time 0.86 min; Figure S26: ESI mass spectra of Streptomycin in the positive ion mode; Figure S27: MS2 spectra of Streptomycin molecular ion m/z 582.2725; Figure S28: MS2 spectra of DP-STR-301 with m/z 302.1233; RT = 1.31 min; detected at a dose of 0.1 kGy; Figure S29: MS2 spectra of DP-STR-289 with m/z 290.1232; RT = 1.82 min; detected at a dose of 0.1 kGy; Figure S30: MS2 spectra of DP-STR-287 with m/z 288.1077; RT = 3.07 min; detected at a dose of 0.1 kGy; Figure S31: TIC chromatogram of Doxycycline, retention time 7.70 min; Figure S32: ESI mass spectra of Doxycycline in the positive ion mode; Figure S33: MS2 spectra of Doxycycline molecular ion m/z 445.1594; Figure S34: TIC chromatogram of Chloramphenicol, retention time 8.73 min; Figure S35: ESI mass spectra of Chloramphenicol in the negative ion mode; Figure S36: MS2 spectra of Chloramphenicol molecular ion m/z 321.0050; Figure S37: Dependence of free energy on the structure of the compound for DP-TC-383; Figure S38: Dependence of free energy on the structure of the compound for DP-TC-415; Figure S39. Dependence of free energy on the structure of the compound for DP-TC-436; Table S1: Physical and chemical properties of antibiotics; Table S2: Hydroxylated decomposition products resulting from the cleavage of the C-H bond and the addition of an OH-group. Reference [42] is cited in the supplementary materials.

## Abbreviations

The following abbreviations are used in this manuscript:

**HPLC-HRMS**	**High-performance liquid chromatography combined with high-resolution mass spectrometry**
DFT	Density functional theory
TC	Tetracycline
DOX	Doxycycline
AMP	Ampicillin
AMO	Amoxicillin
PENG	Penicillin G (Benzylpenicillin)
STR	Streptomycin
CAP	Chloramphenicol
DPs	Degradation products

## Appendix A

**Table A1:** HPLC-MS analysis conditions.

HPLC-MS Parameters	Value
HPLC	
Column	Thermo RS LC HPLC (150 mm × 2.1 mm, 2.2 µm particle size)
Mobile phases	Mobile phase *A*: 0.1% formic acid in water Mobile phase *B*: 100% acetonitrile
Flow rate	0.3 mL/min
Column temperature	35 °C
Injection volume	20 µL
Gradient elution mode	0–2 min—5% B; 2–15 min—95% B; 15–18 min—95% B; 18–19 min—5% B; 19–24 min—5% B.
MS	
Ionization method	Electrospray ionization (ESI)
Ion generation mode	Both polarities (+ and −)
Ion source temperature	325 °C
Drying gas pressure	344.7 kPa
Nebulizing gas pressure	69 kPa
Capillary voltage	+3500; −2500 V
Mass scanning range	100–1000 *m*/*z*
