# Supplementary material for: Electron Beam Irradiation for Efficient Antibiotic Degradation in Aqueous Solutions
Source: Antibiotics (Basel). 2025 Aug 15;14(8):833. doi: 10.3390/antibiotics14080833 (PMC12382617; doi:10.3390/antibiotics14080833)
Supplement: Supplementary file 1 [file antibiotics-14-00833-s001.zip › antibiotics-3801647_supplementary.pdf]

Supplementary Materials

*Corresponding author: E-mail: opruneko_anastasiya@mail.ru

Tetracycline

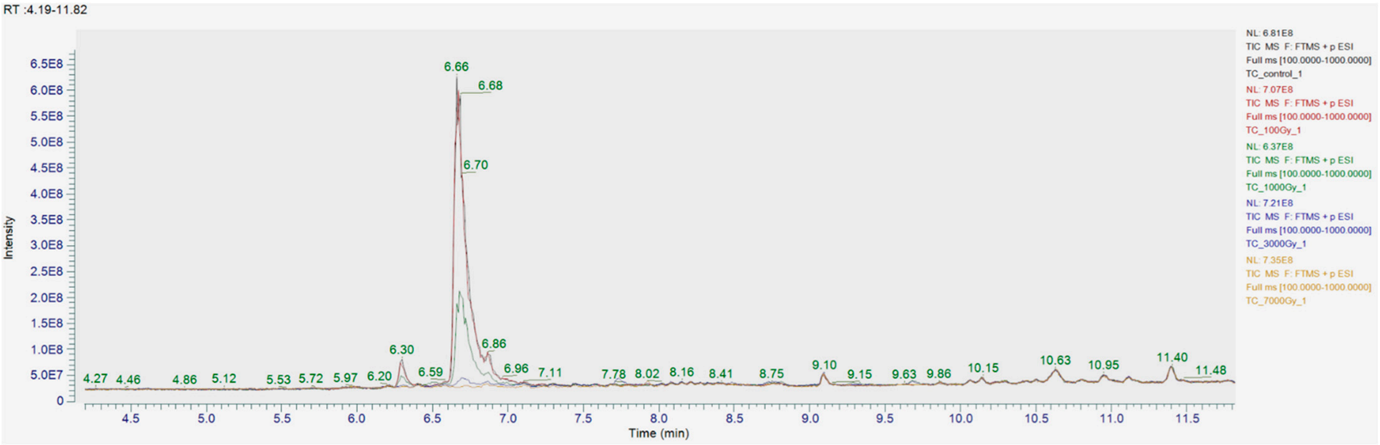

Figure S1. TIC chromatogram of Tetracycline, retention time(RT) 6.68 min.

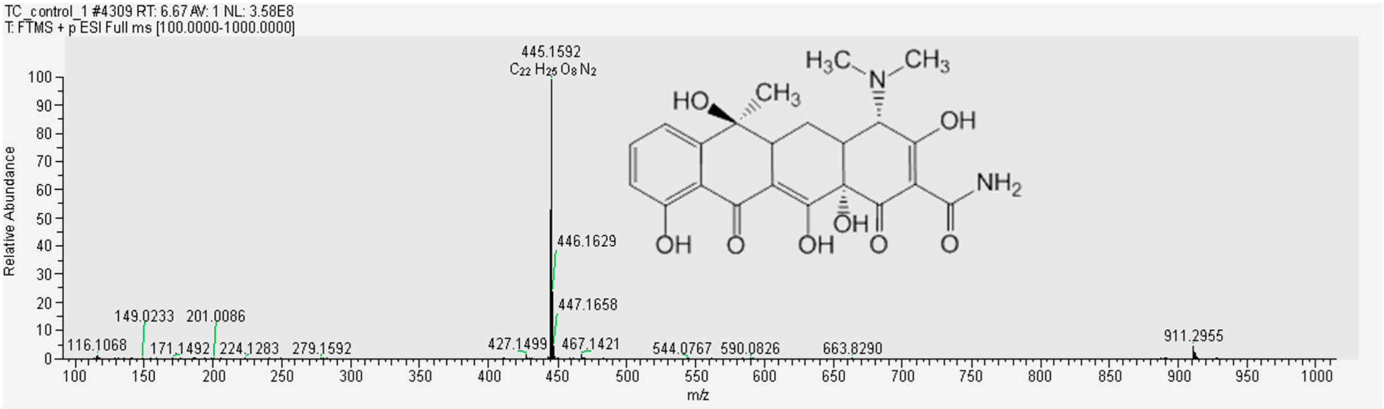

Figure S2. ESI mass spectra of Tetracycline in the positive ion mode

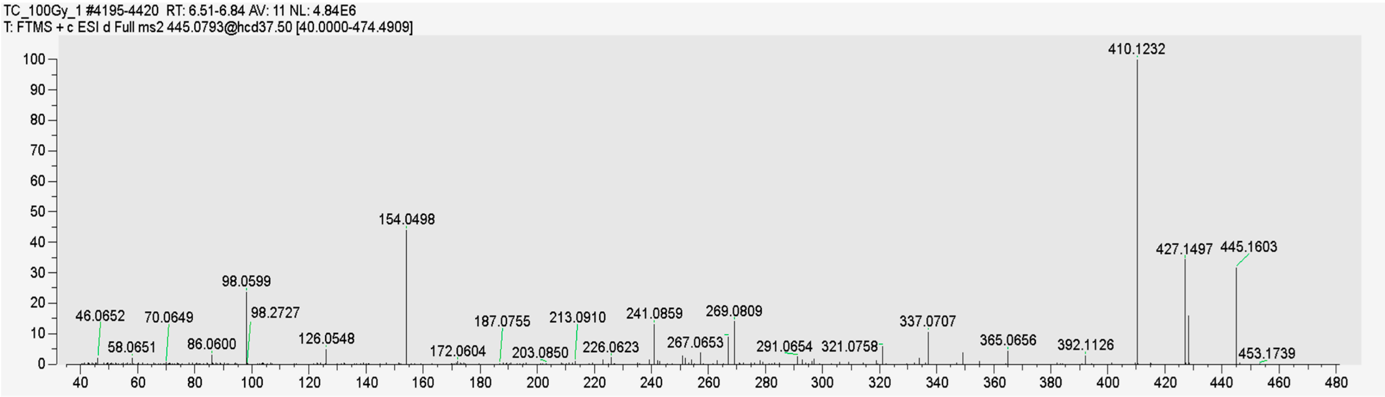

Figure S3. MS2 spectra of Tetracycline molecular ion m/z 445.1592

DP-TC-460

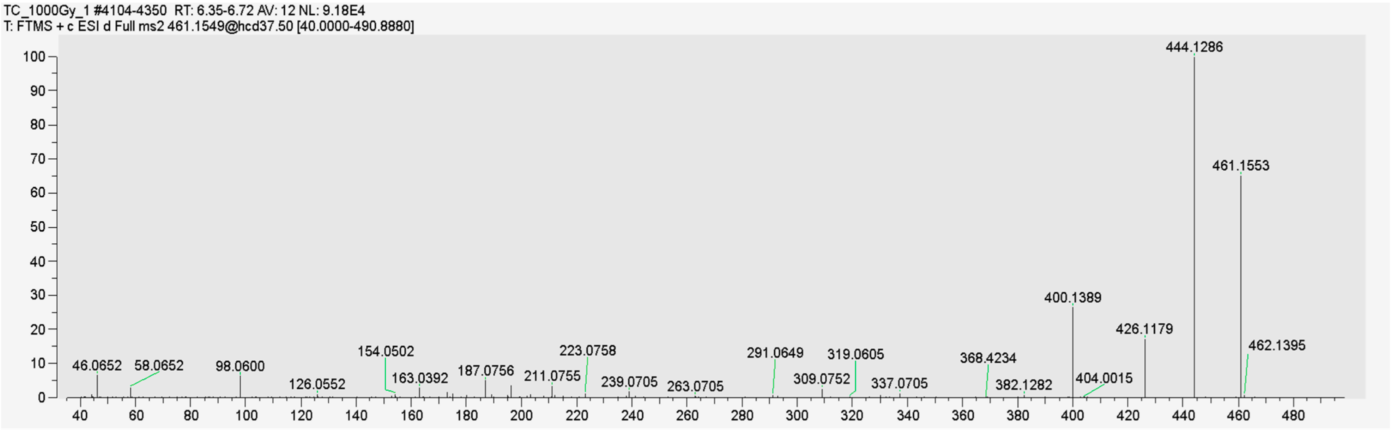

Figure S4. MS2 spectra of DP-TC-460 with m/z 461.1549; RT=6.51 min; detected at a dose of 1 kGy

DP-TC-399

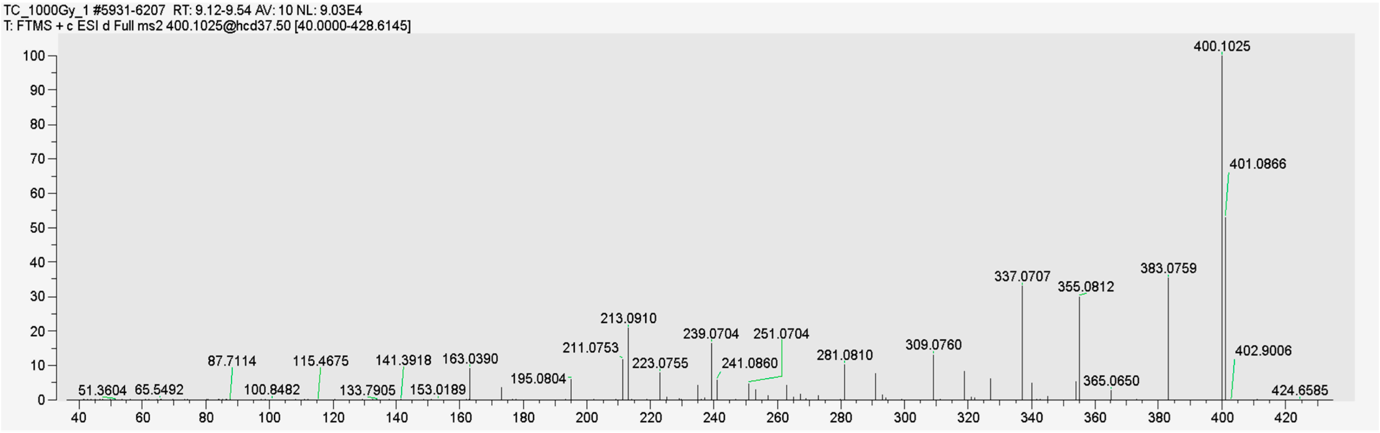

Figure S5. MS2 spectra of DP-TC-399 with m/z 400.1024; RT=9.33 min; detected at a dose of 1 kGy

DP-TC-383

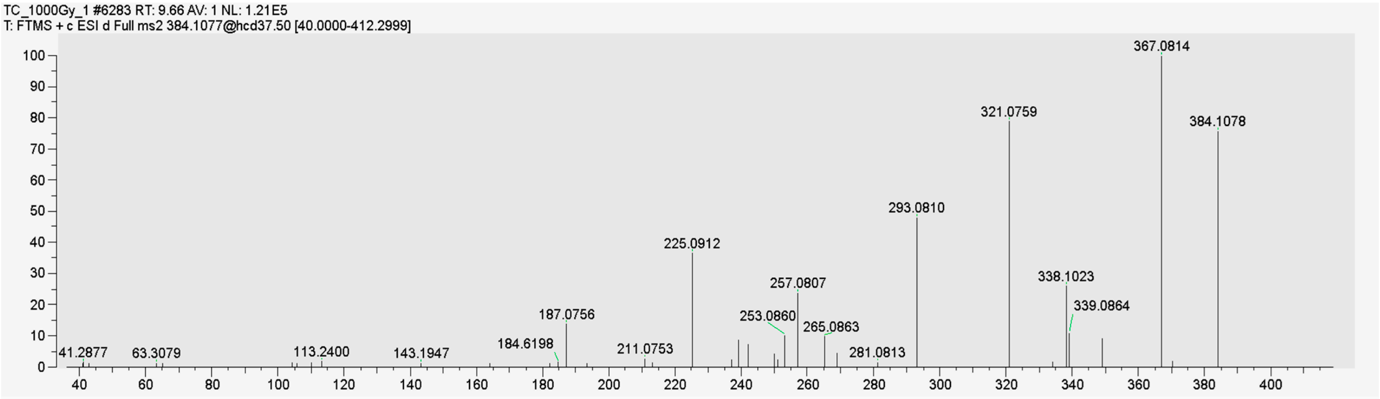

Figure S6. MS2 spectra of DP-TC-383 with m/z 384.1074; RT=9.68 min; detected at a dose of 1 kGy

DP-TC-415

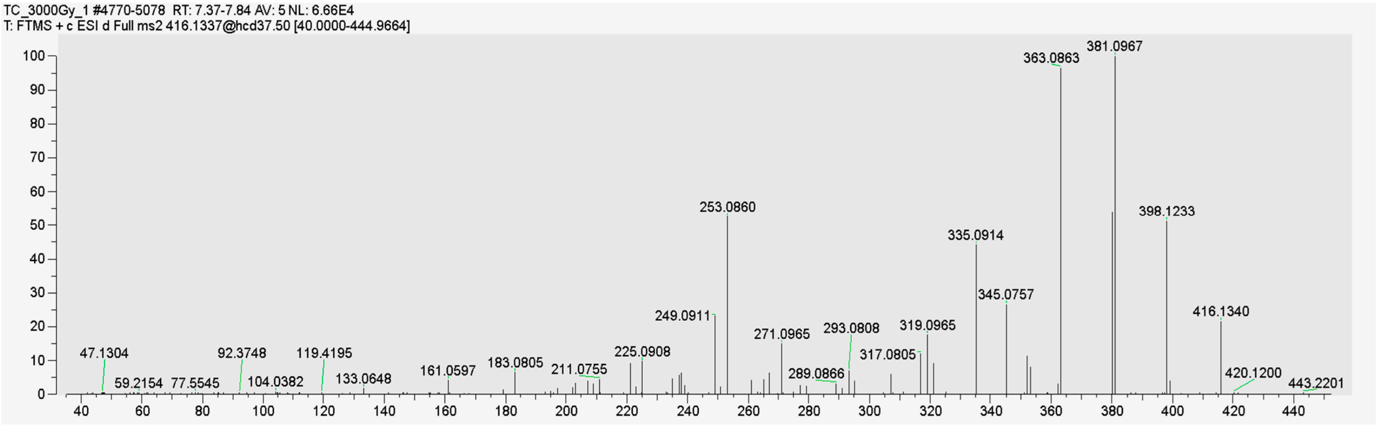

Figure S7. MS2 spectra of DP-TC-415 with m/z 416.1334; RT=7.74 min; detected at a dose of 1 kGy

DP-TC-436

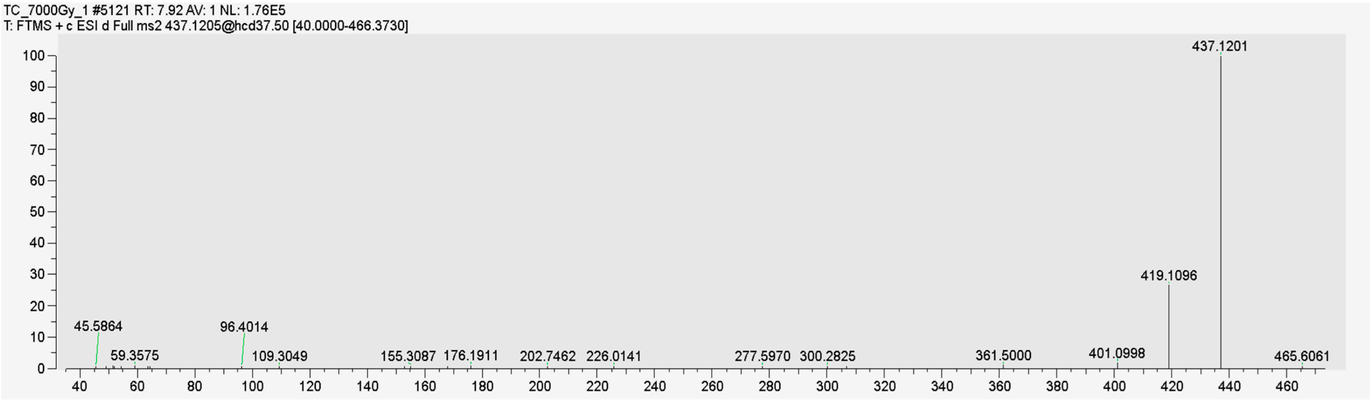

Figure S8. MS2 spectra of DP-TC-436 with m/z 437.1205; RT=7.92 min; detected at a dose of 3 kGy

Amoxicillin

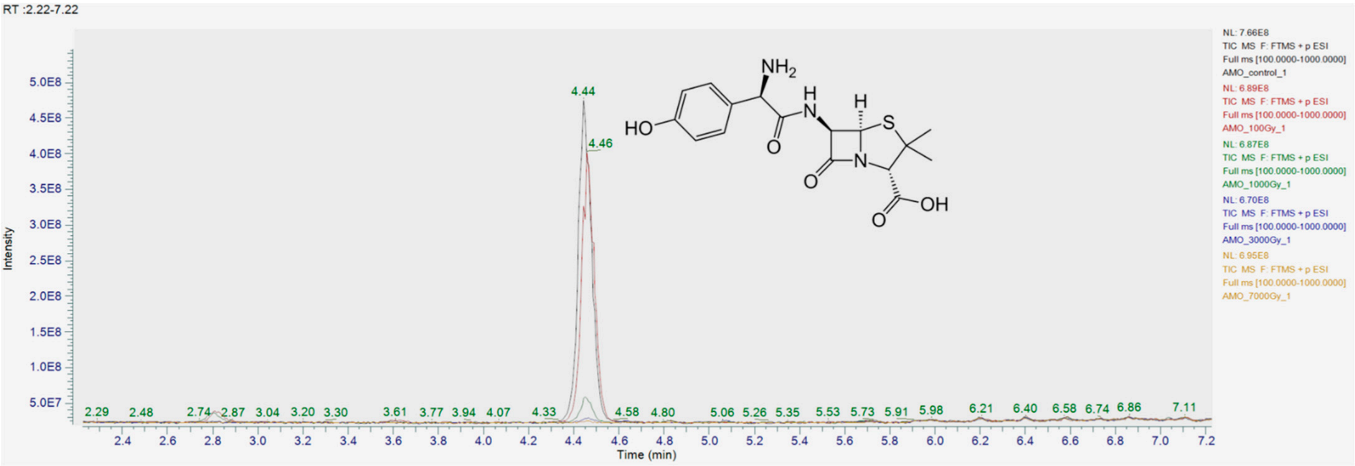

Figure S9. TIC chromatogram of Amoxicillin, retention time 4.45 min.

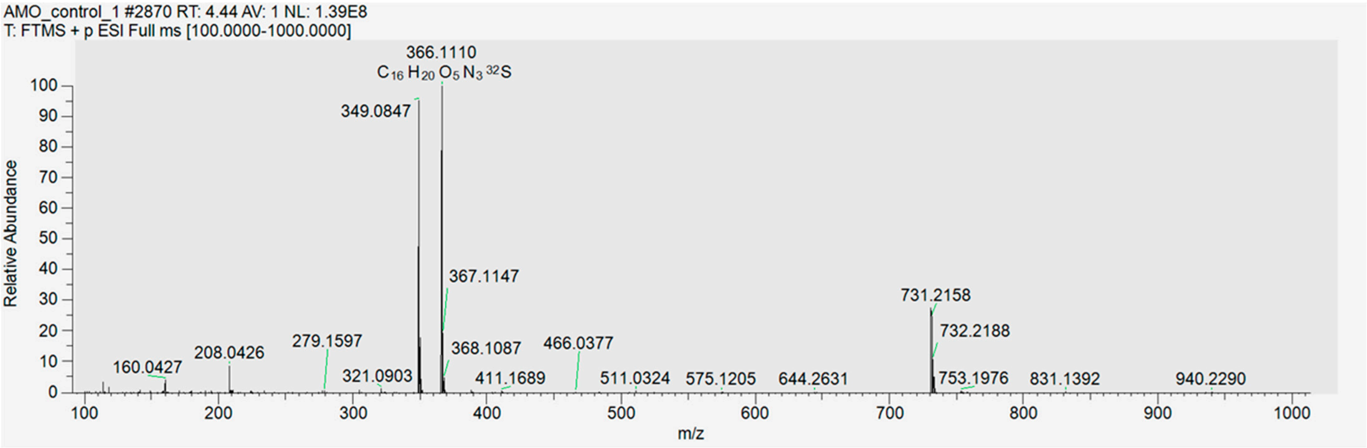

Figure S10. ESI mass spectra of Amoxicillin in the positive ion mode

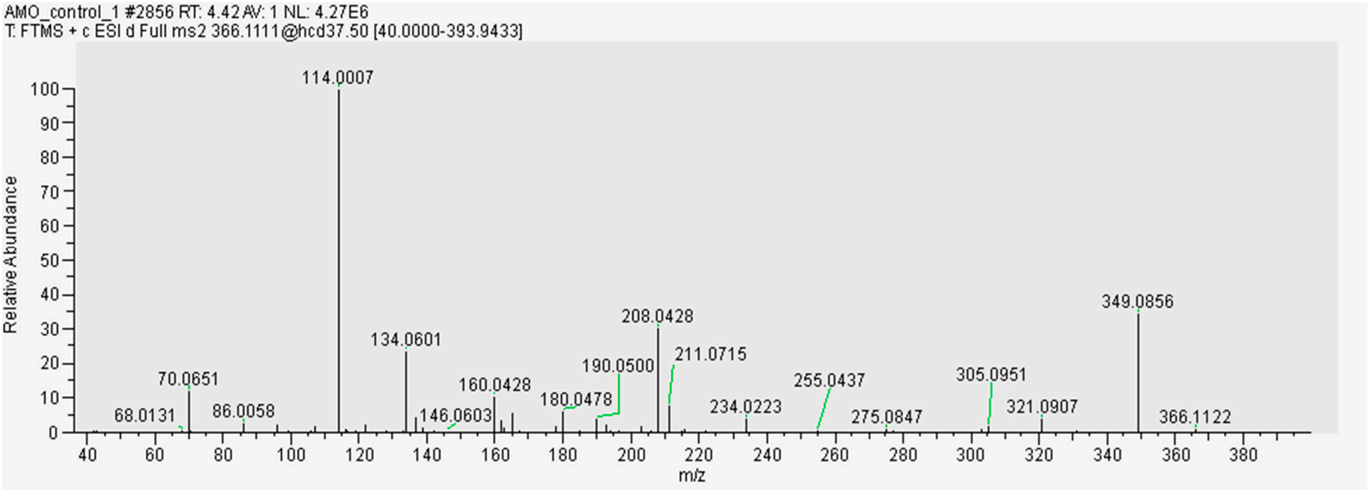

Figure S11. MS2 spectra of Amoxicillin molecular ion m/z 366.1110

DP-AMO-364

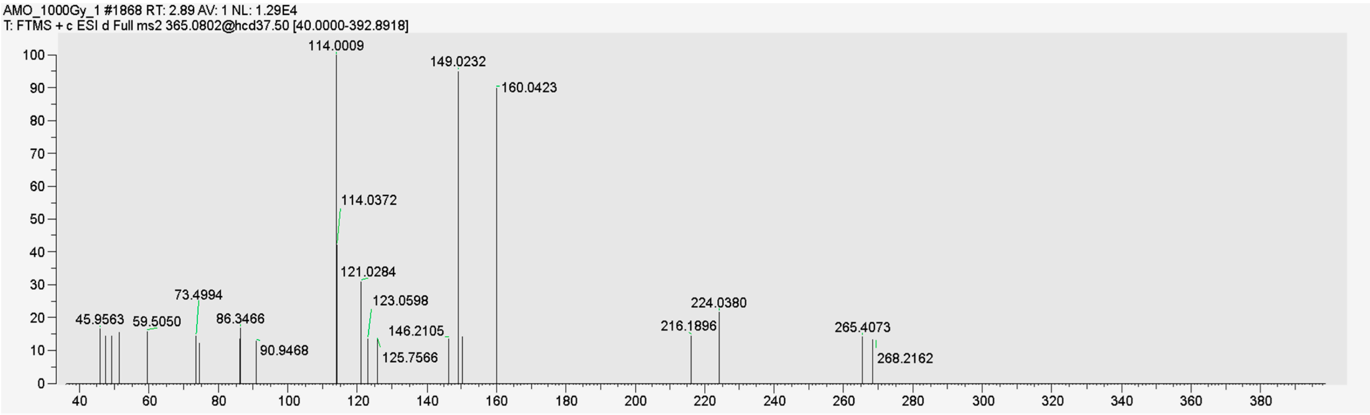

Figure S12. MS2 spectra of DP-AMO-364 with m/z 365.0801; RT=2.81 min; detected at a dose of 0.1 kGy

DP-AMO-381

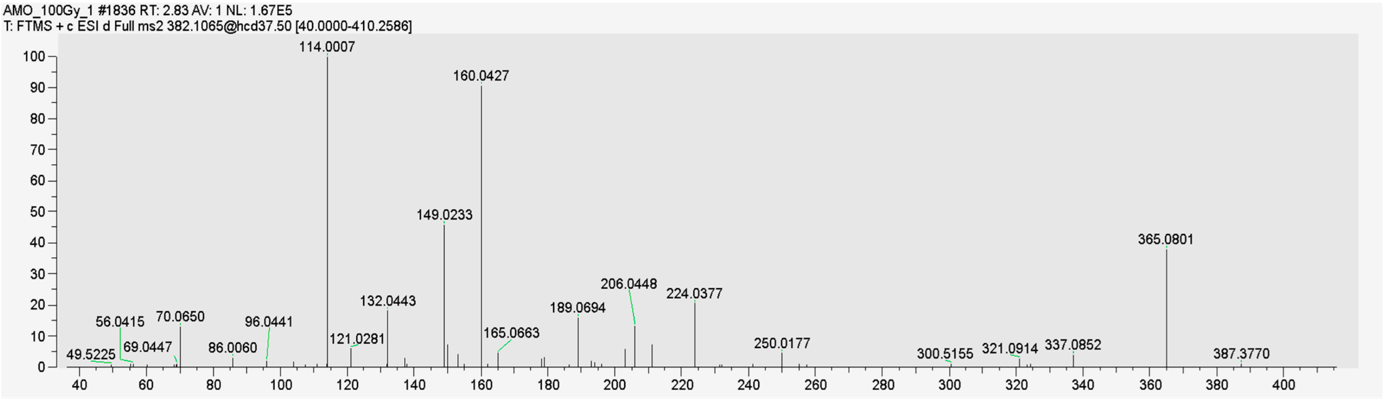

Figure S13. MS2 spectra of DP-AMO-381 with m/z 382.1066; RT=1.97 min; detected at a dose of 0.1 kGy

Ampicillin

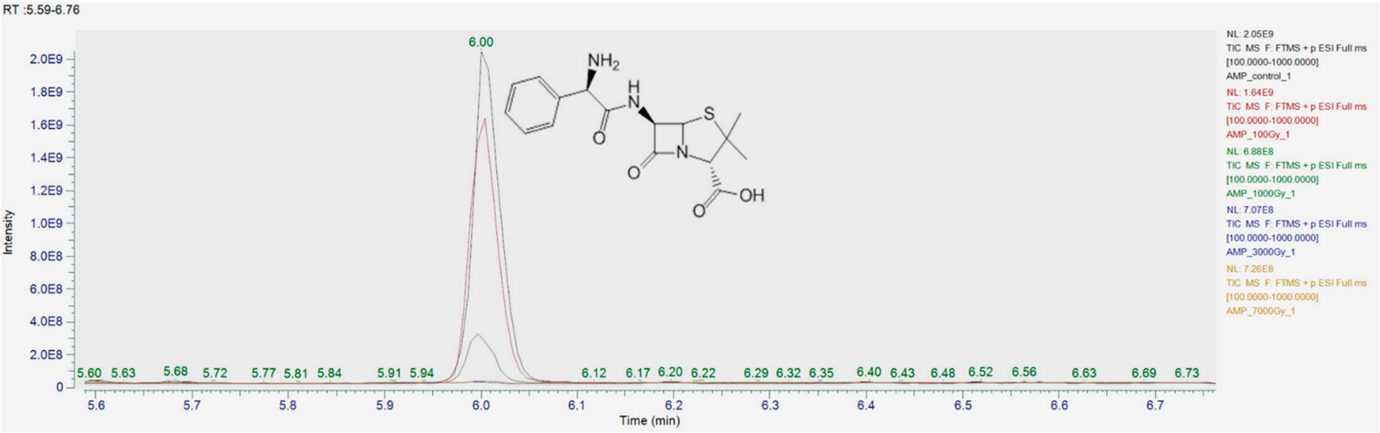

Figure S14. TIC chromatogram of Ampicillin, retention time 6.00 min.

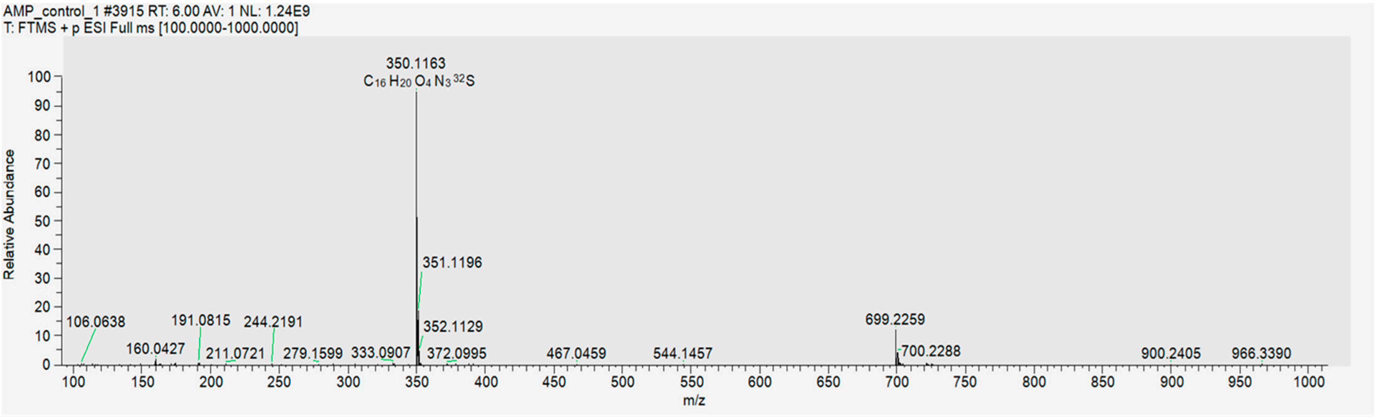

Figure S15. ESI mass spectra of Ampicillin in the positive ion mode

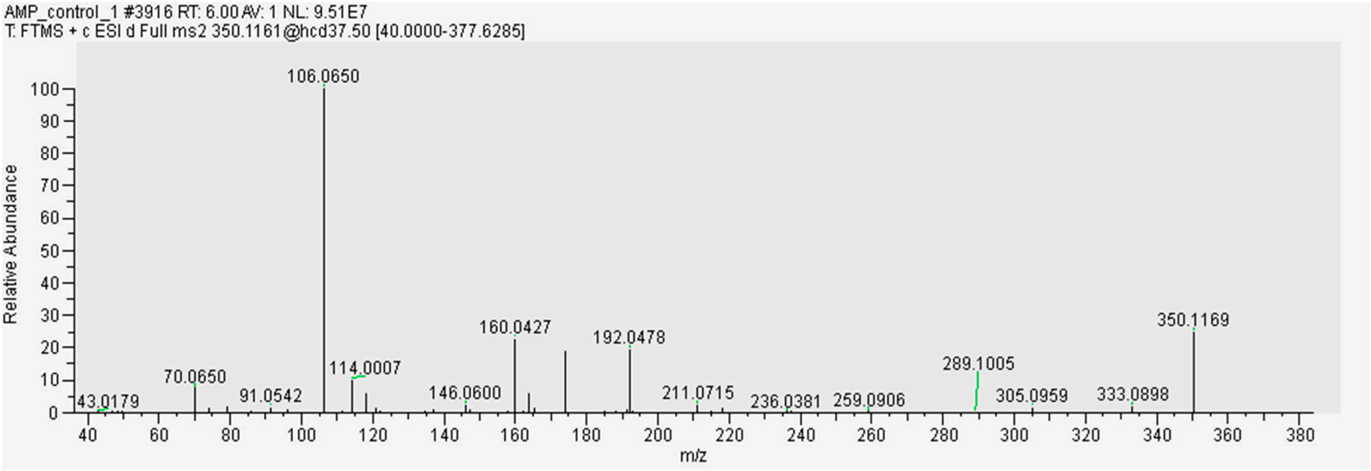

Figure S16. MS2 spectra of Ampicillin molecular ion m/z 350.1163

DP-AMP-365

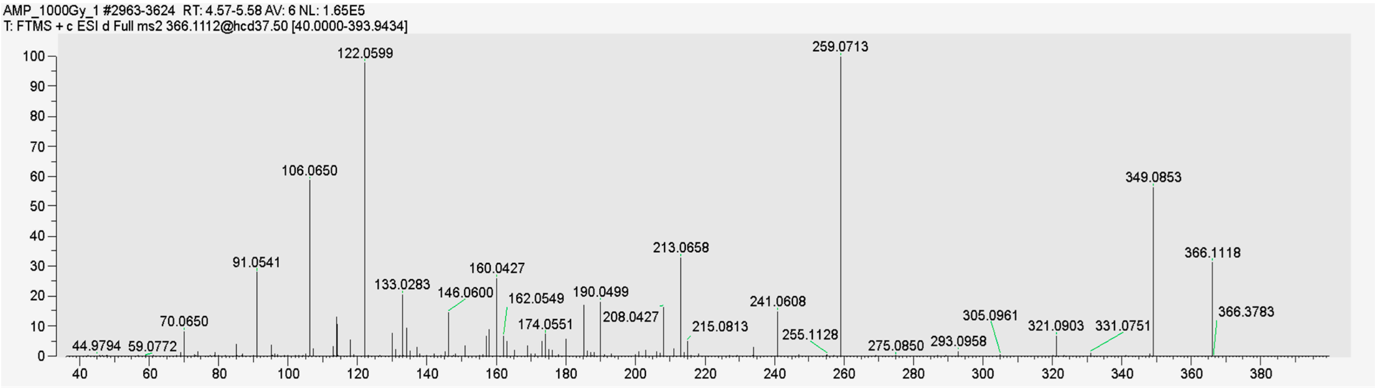

Figure S17. MS2 spectra of DP-AMP-365 with m/z 366.1111; RT=5.12 min; detected at a dose of 0.1 kGy

DP-AMP-367

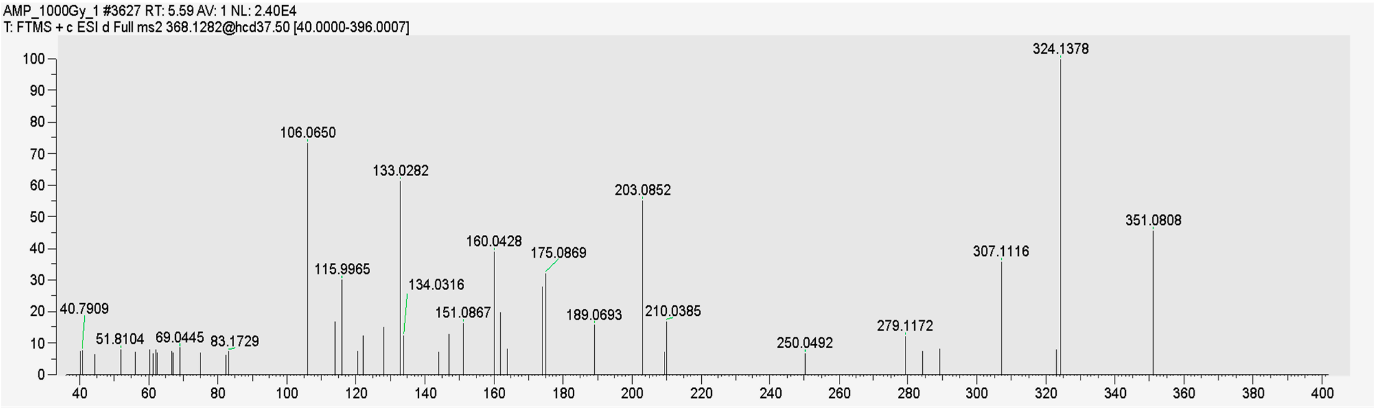

Figure S18. MS2 spectra of DP-AMP-367 with m/z 368.1274; RT=5.60 min; detected at a dose of 0.1 kGy

Benzylpenicillin (Penicillin G)

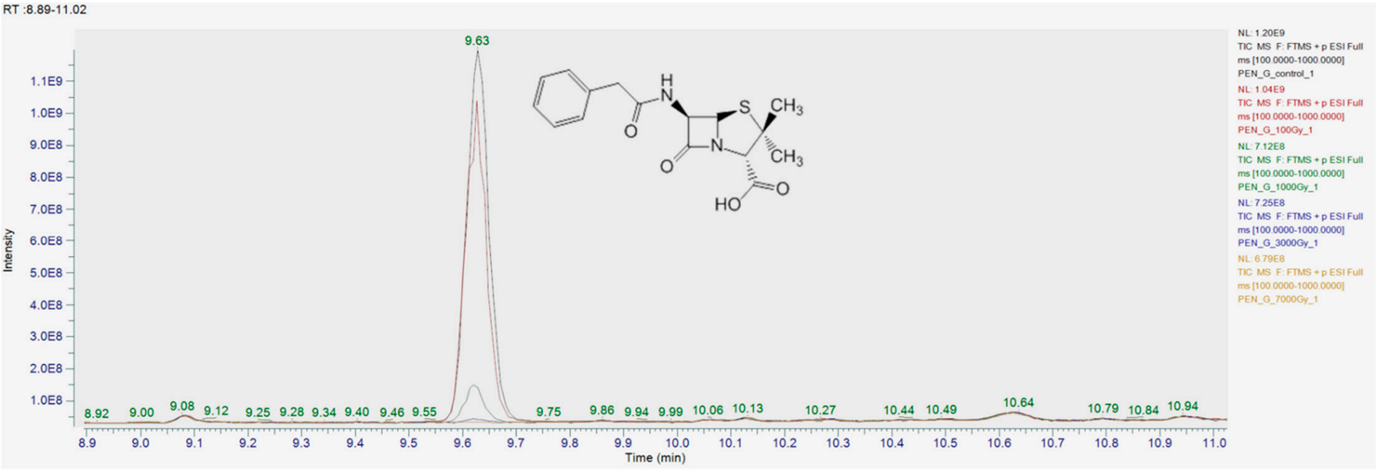

Figure S19. TIC chromatogram of Benzylpenicillin, retention time 9.62 min.

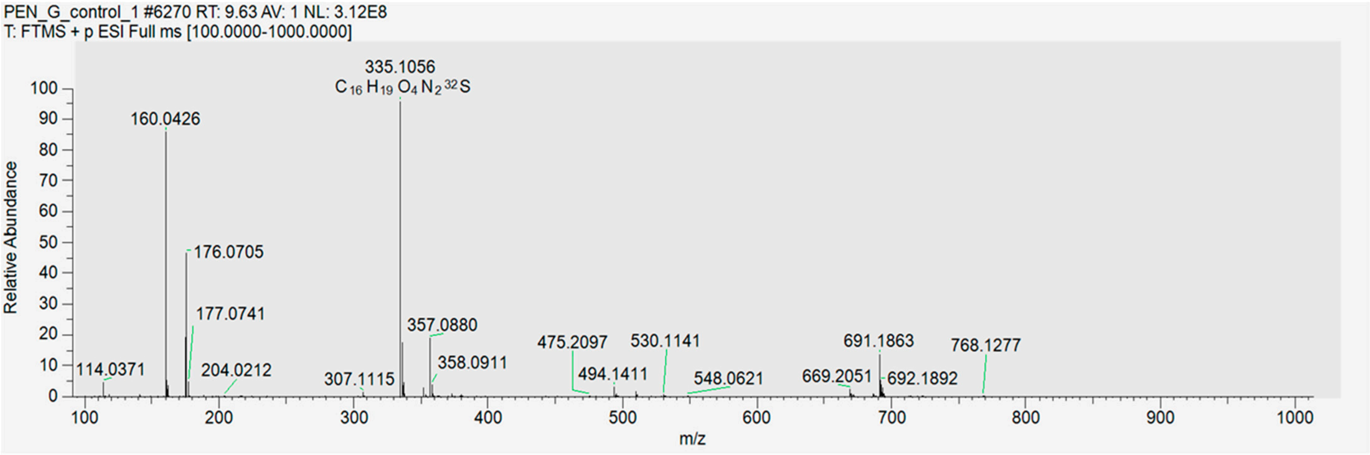

Figure S20. ESI mass spectra of Benzylpenicillin in the positive ion mode

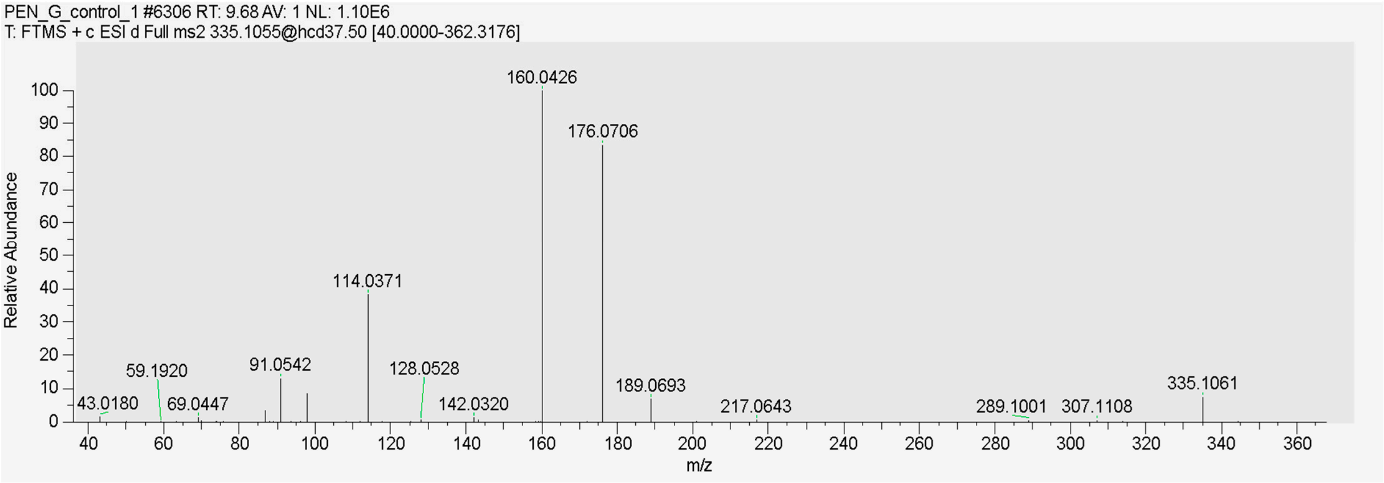

Figure S21. MS2 spectra of Benzylpenicillin molecular ion m/z 335.1056

DP-PENG-350

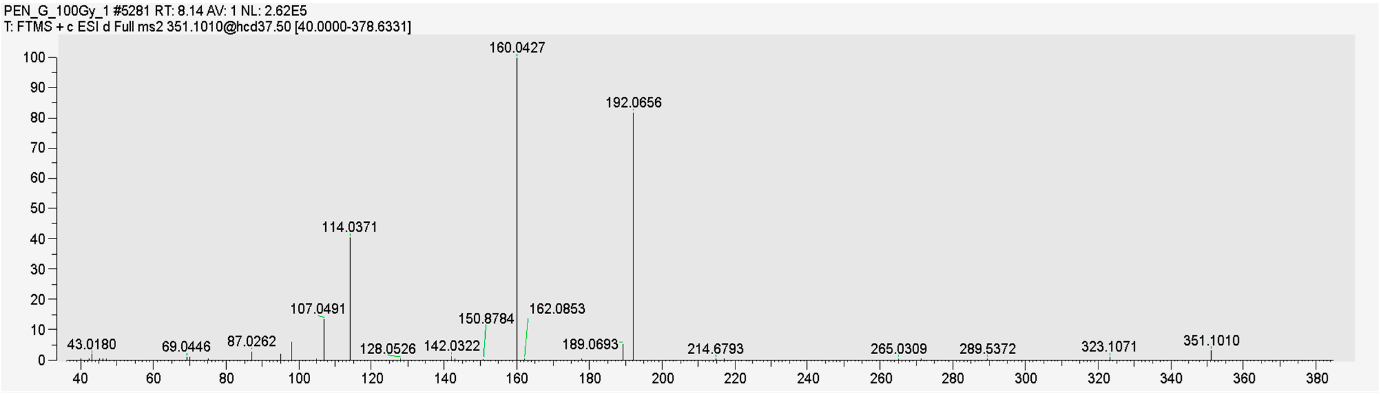

Figure S22. MS2 spectra of DP-PENG-350 with m/z 351.1007; RT=8.16; 8.40; 8.83 min; detected at a dose of 0.1 kGy

DP-PENG-352

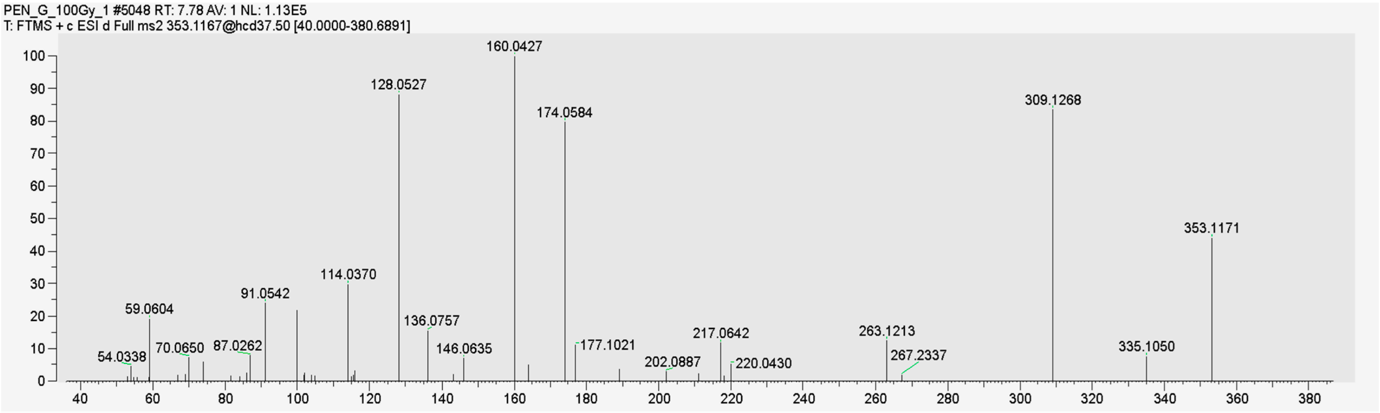

Figure S23. MS2 spectra of DP-PENG-352 with m/z 353.1168; RT=7.83 min; detected at a dose of 0.1 kGy

DP-PENG-308

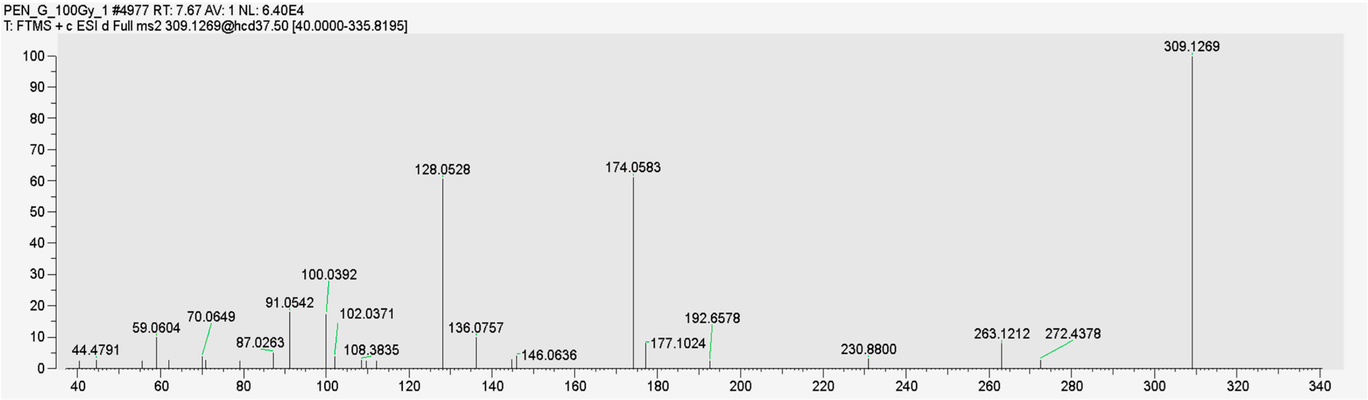

Figure S24. MS2 spectra of DP-PENG-308 with m/z 309.1276; RT=7.83 min; detected at a dose of 0.1 kGy

Streptomycin

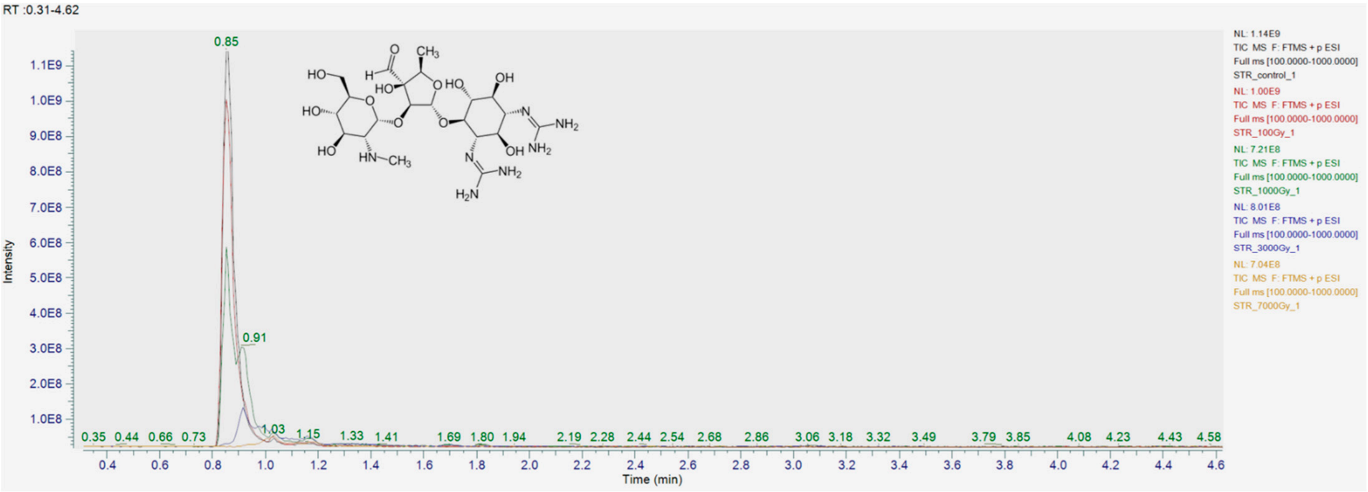

Figure S25. TIC chromatogram of Streptomycin, retention time 0.86 min.

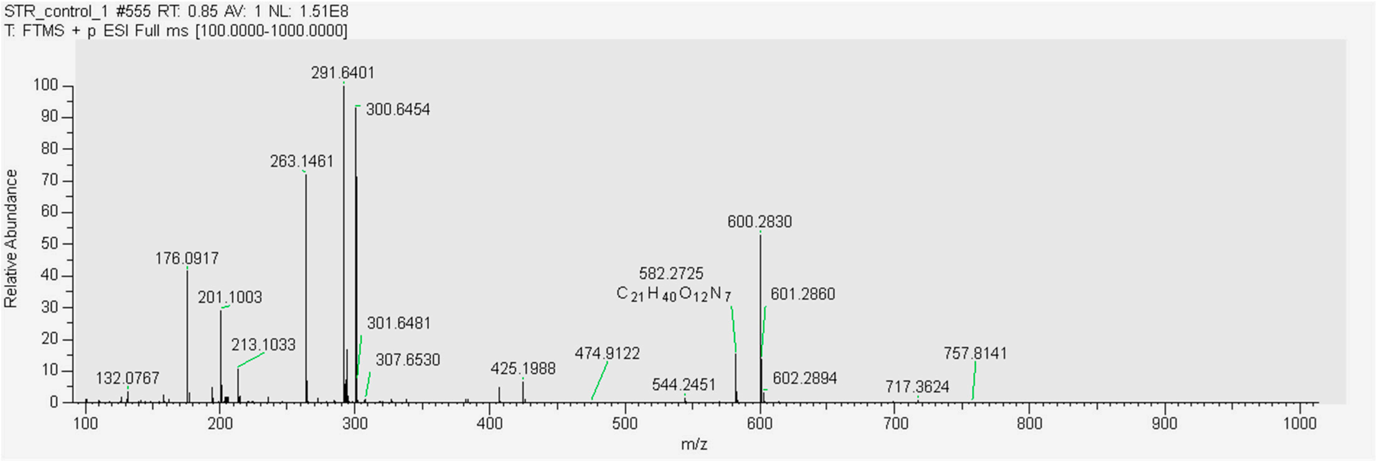

Figure S26. ESI mass spectra of Streptomycin in the positive ion mode

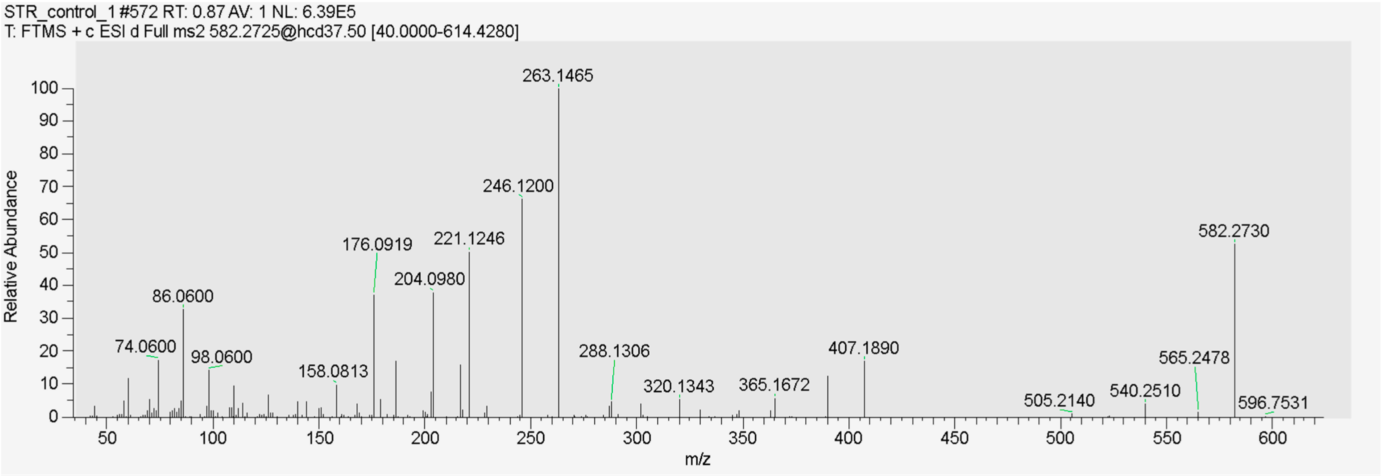

Figure S27. MS2 spectra of Streptomycin molecular ion m/z 582.2725

DP-STR-301

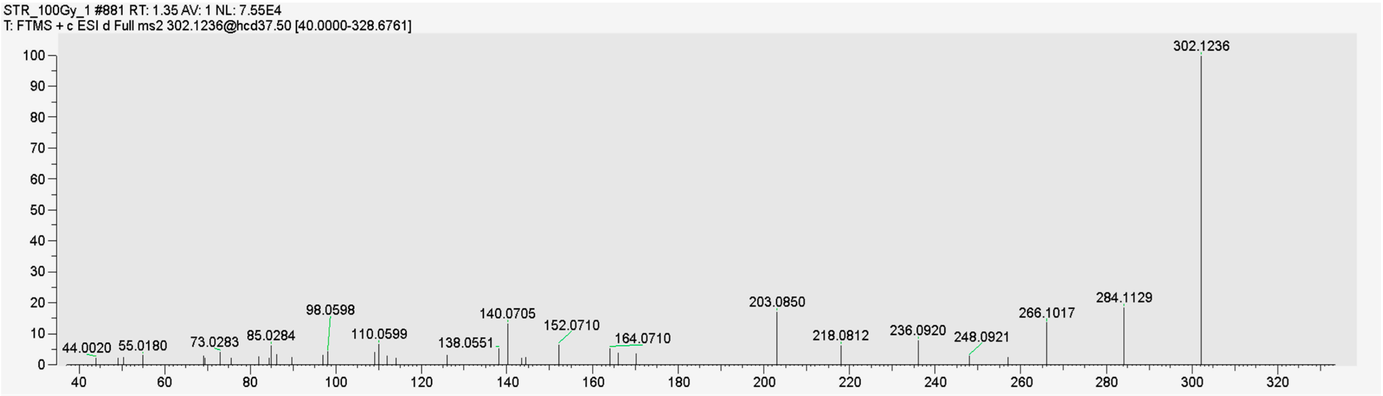

Figure S28. MS2 spectra of DP-STR-301 with m/z 302.1233; RT=1.31 min; detected at a dose of 0.1 kGy

DP-STR-289

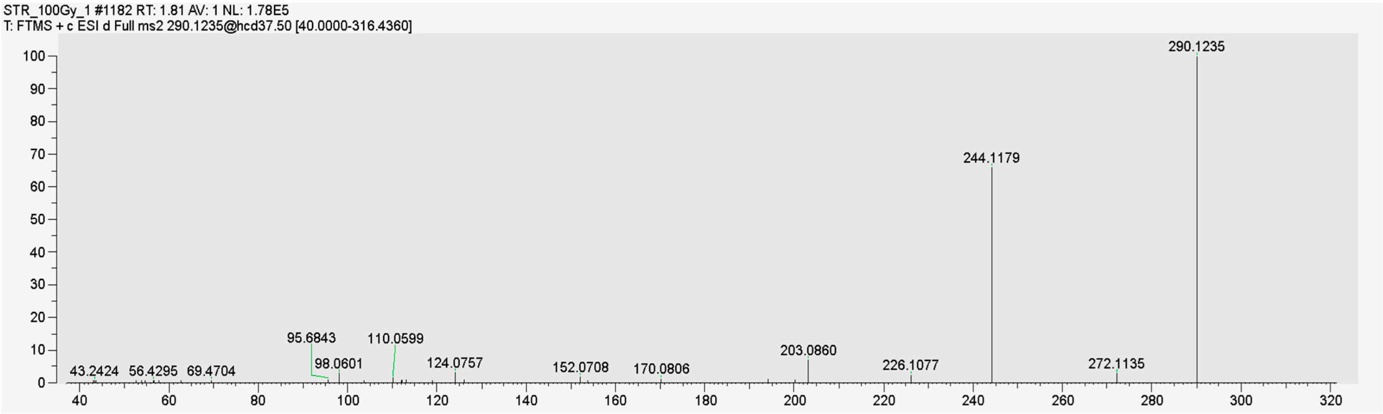

Figure S29. MS2 spectra of DP-STR-289 with m/z 290.1232; RT=1.82 min; detected at a dose of 0.1 kGy

DP-STR-287

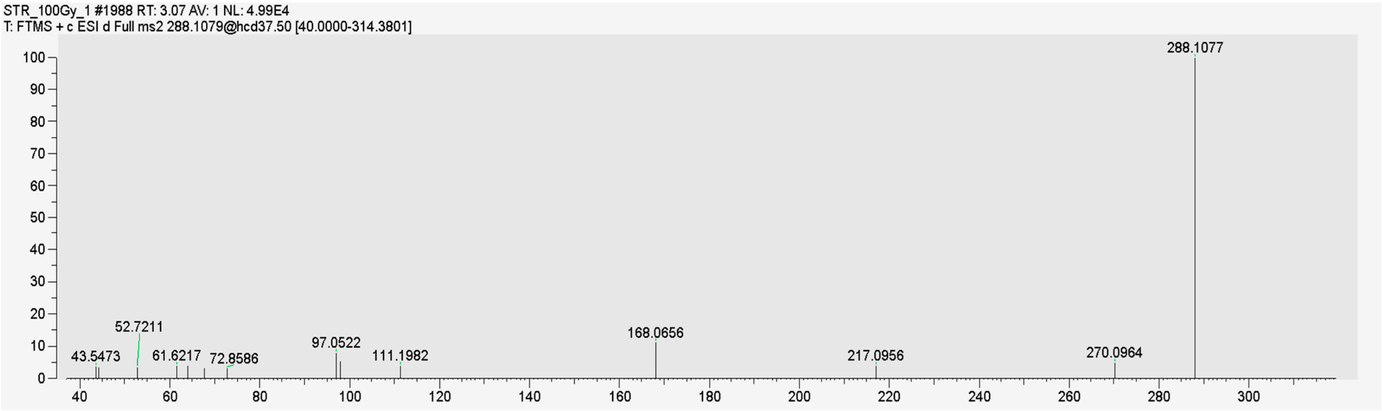

Figure S30. MS2 spectra of DP-STR-287 with m/z 288.1077; RT=3.07 min; detected at a dose of 0.1 kGy

Doxycycline

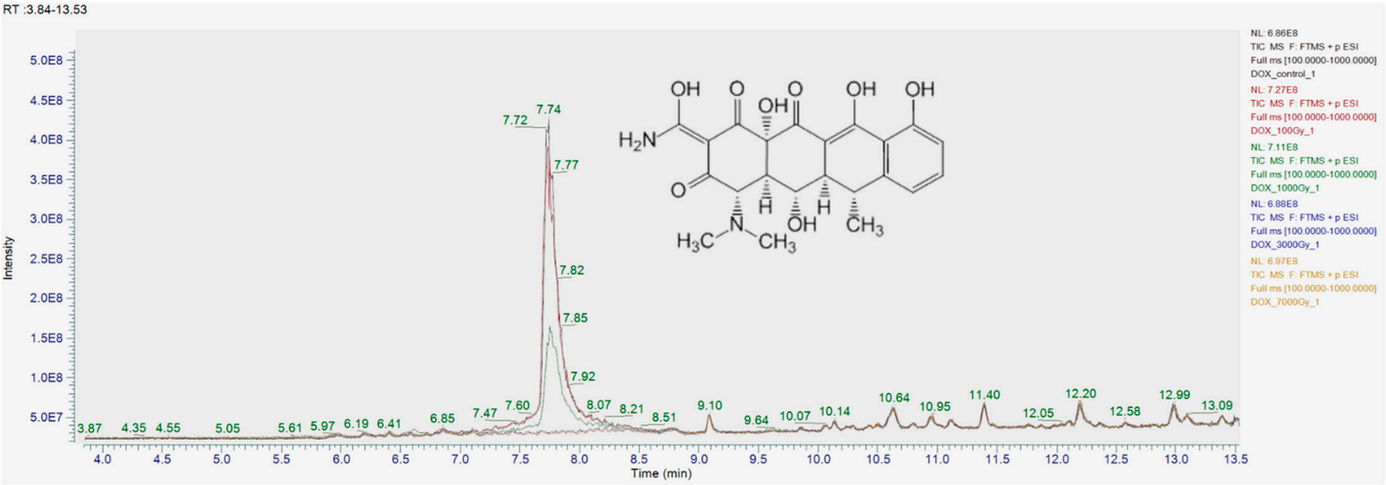

Figure S31. TIC chromatogram of Doxycycline, retention time 7.70 min.

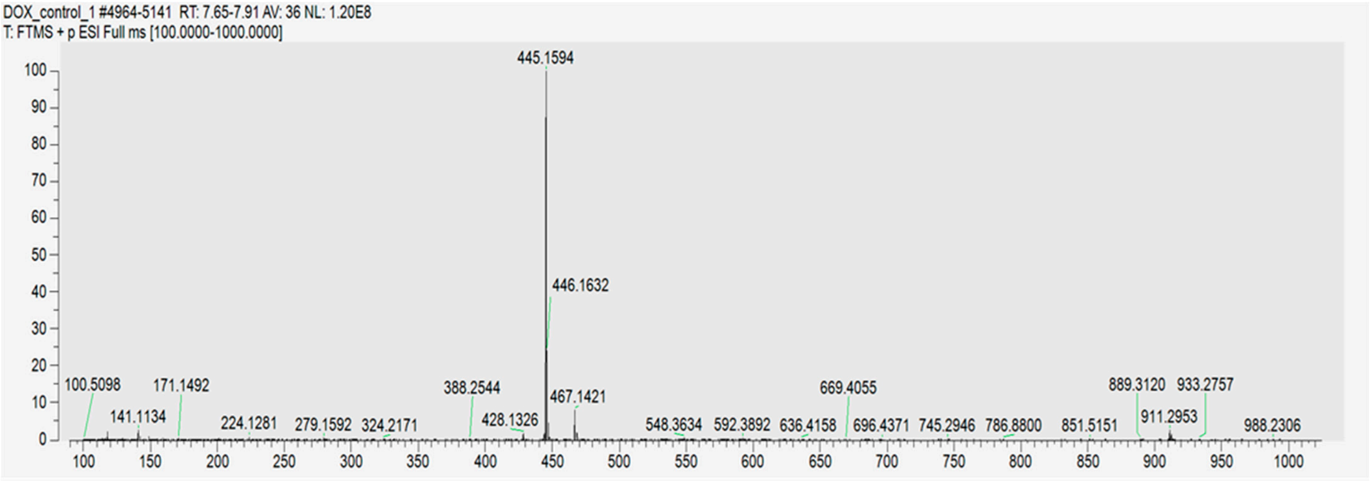

Figure S32. ESI mass spectra of Doxycycline in the positive ion mode

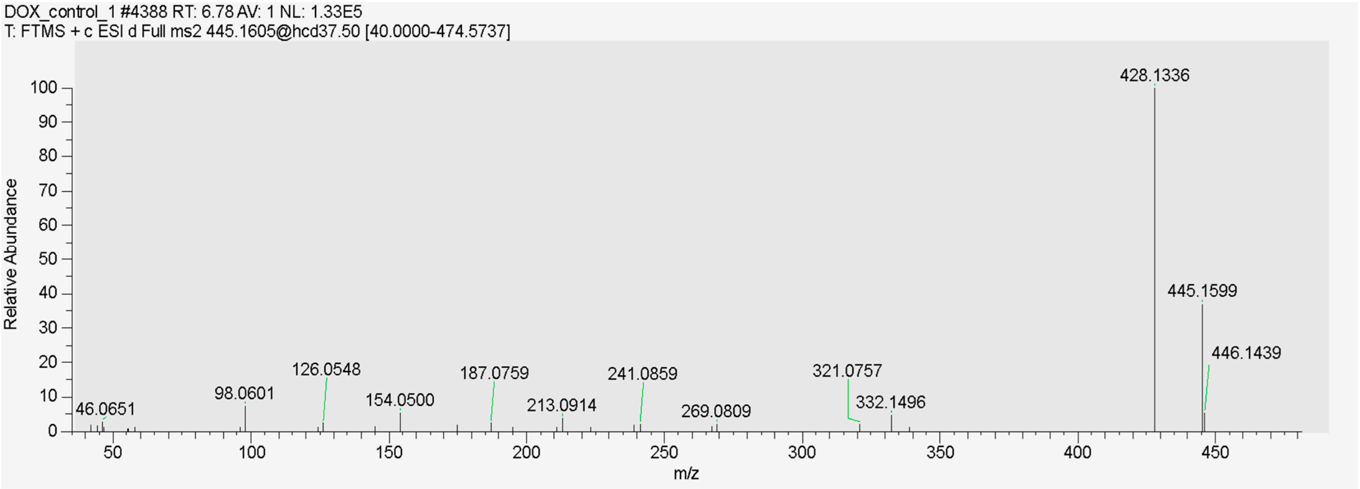

Figure S33. MS2 spectra of Doxycycline molecular ion m/z 445.1594

Chloramphenicol

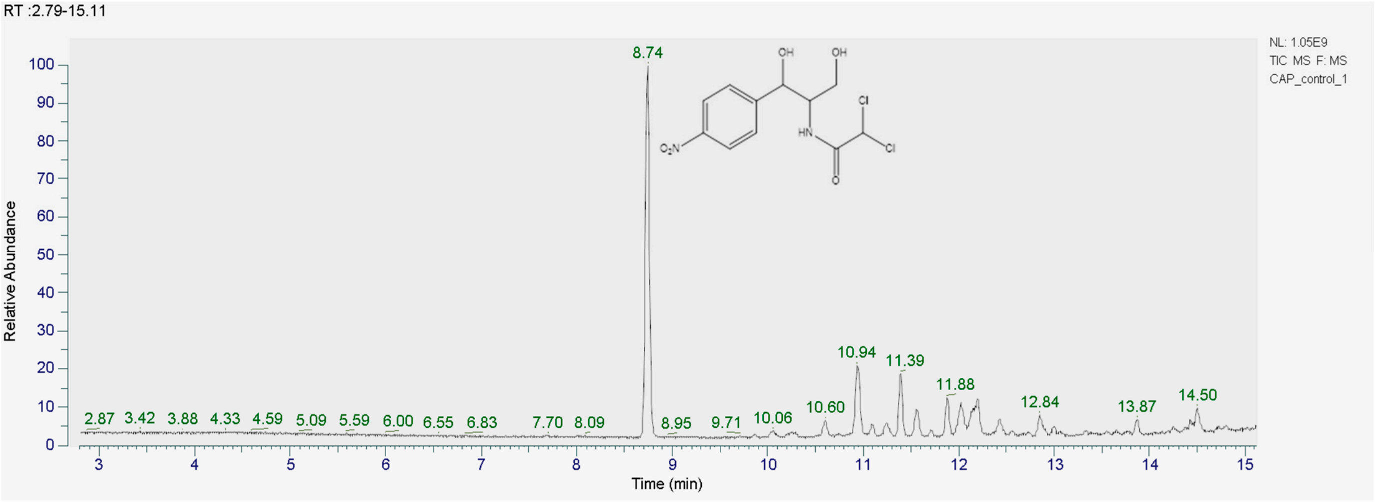

Figure S34. TIC chromatogram of Chloramphenicol, retention time 8.73 min

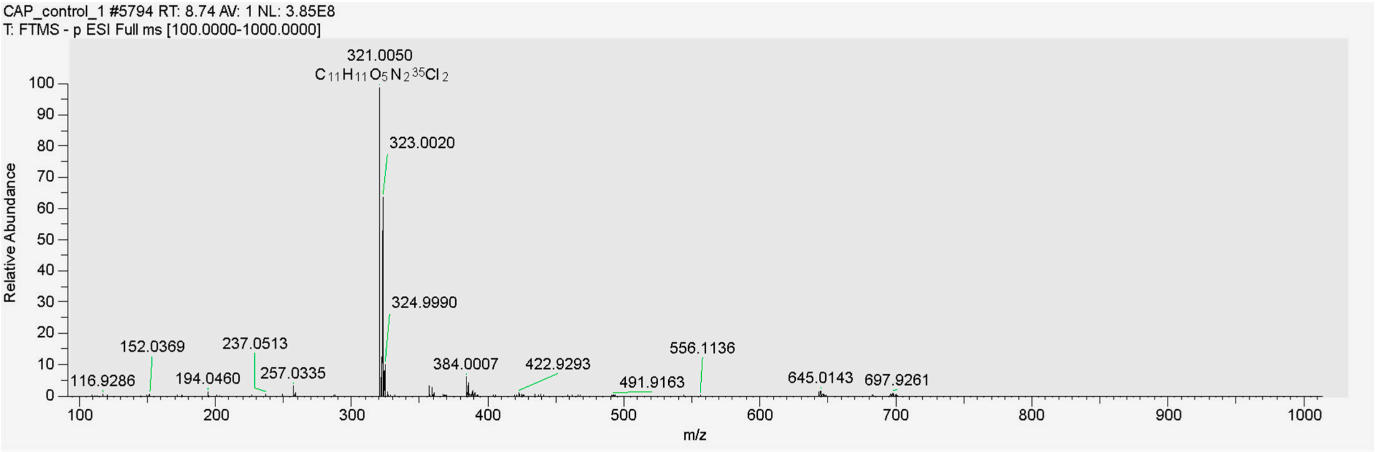

Figure S35. ESI mass spectra of Chloramphenicol in the negative ion mode

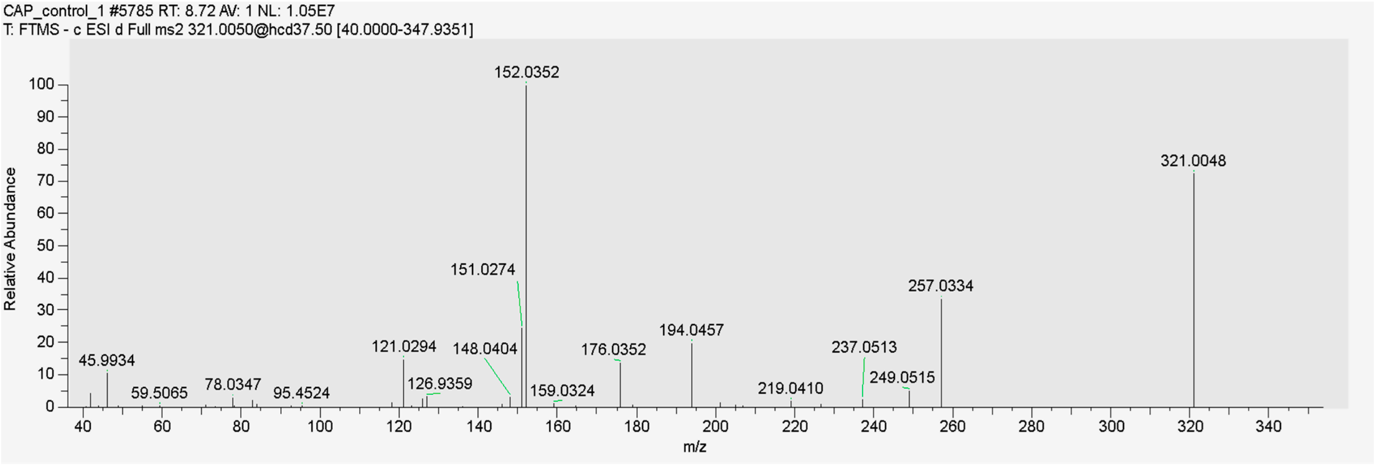

Figure S36. MS2 spectra of Chloramphenicol molecular ion m/z 321.0050

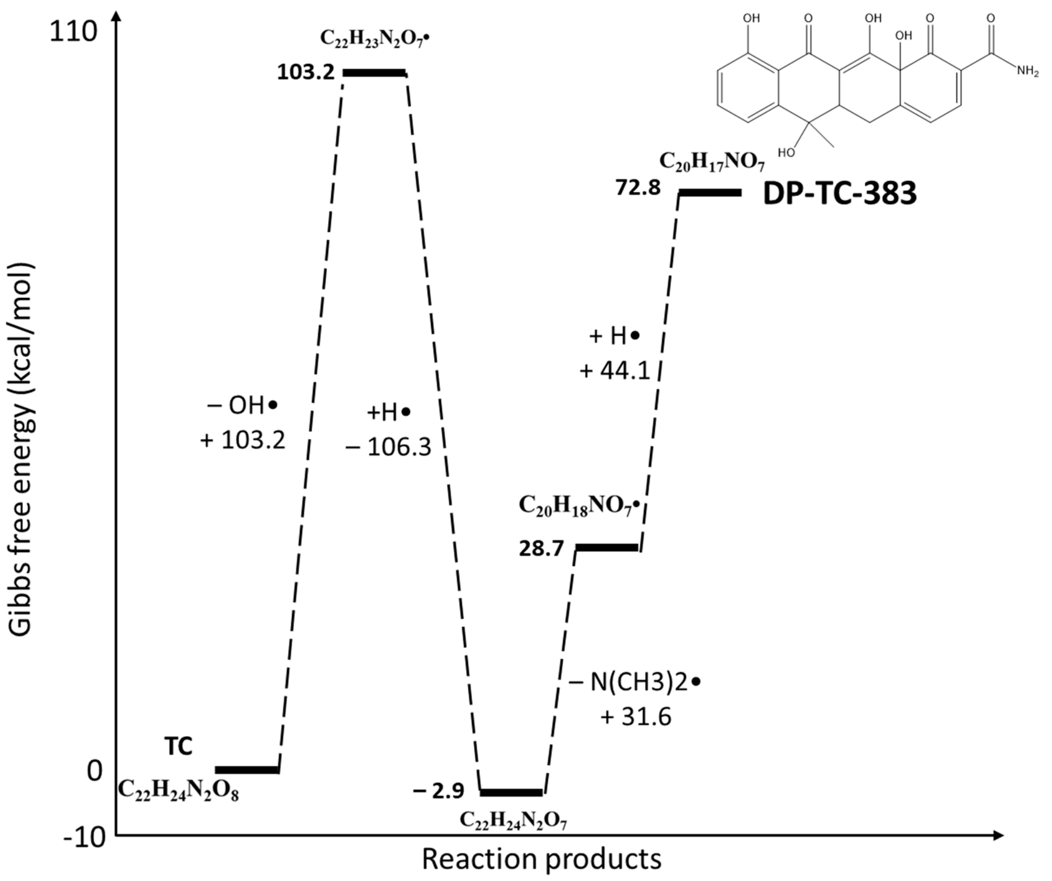

Figure S37. Dependence of free energy on the structure of the compound for DP-TC-383

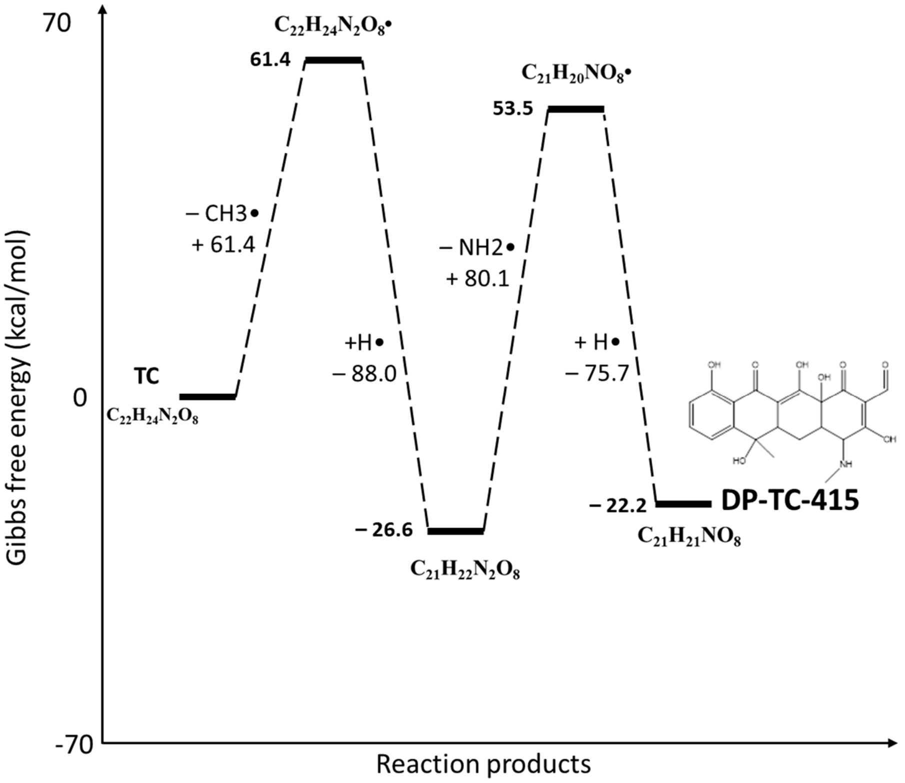

Figure S38. Dependence of free energy on the structure of the compound for DP-TC-415

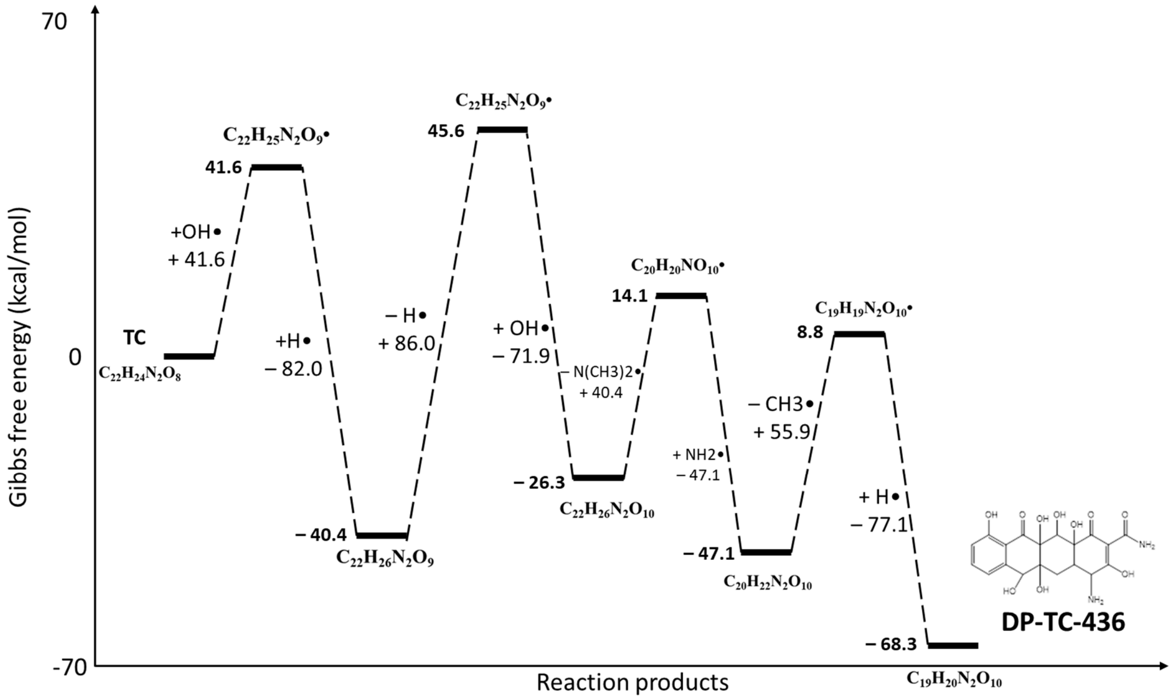

Figure S39. Dependence of free energy on the structure of the compound for DP-TC-436

Table S1. Physical and chemical properties of antibiotics

Compound	Structure	Formula	Molecular mass (g/mol)	Solubility (mg/mL) (25°C) [42]
Tetracycline	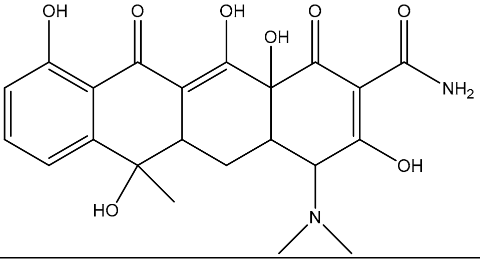	$C_{22}H_{24}N_2O_8$	444.1538	231
Doxycycline	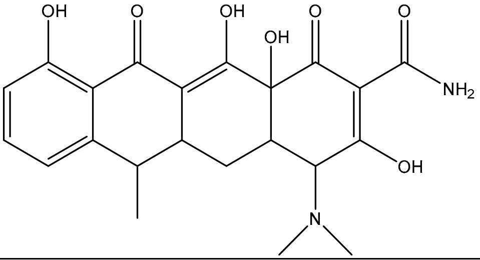	$C_{22}H_{24}N_2O_8$	444.1538	76.9
Ampicillin	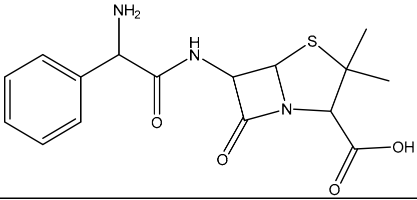	$C_{16}H_{19}N_3O_4S$	349.1096	10,100
Amoxicillin	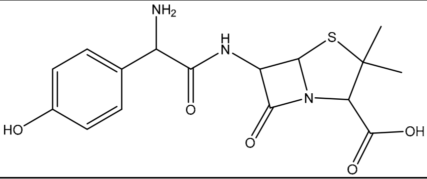	$C_{16}H_{19}N_3O_5S$	365.1045	3430
Benzylpenicillin (Penicillin G)	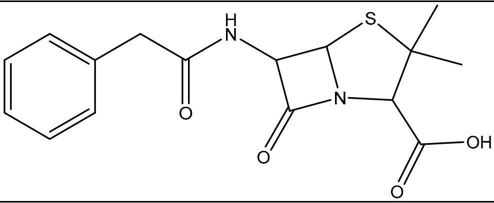	$C_{16}H_{18}N_2O_4S$	334.0987	210
Streptomycin	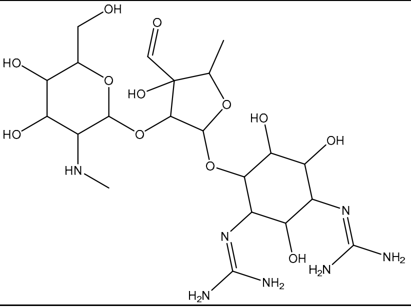	$C_{21}H_{39}N_7O_{12}$	581.2656	89,000

Chloramphenicol	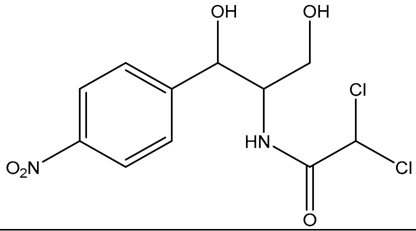	$C_{11}H_{12}N_2O_5Cl_2$	322.0123	2500

Table S2. Hydroxylated decomposition products resulting from the cleavage of the C-H bond and the addition of an OH-group

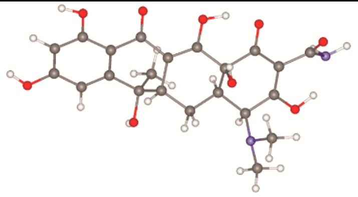<br>Nastya-1<br>**C5**<br>E= -1637,861726; G=236.0;<br>ΔE=0; ΔG=0;	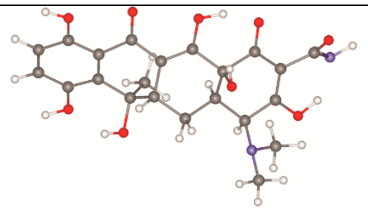<br>Nastya-3<br>**C4**<br>E= -1637,860401; G=237.0;<br>ΔE=0.8; ΔG=1.8;	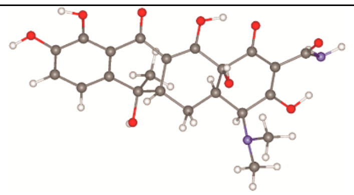<br>Nastya-2<br>**C6**<br>E= -1637,861594; G=236.0;<br>ΔE=0.1; ΔG=0.1;
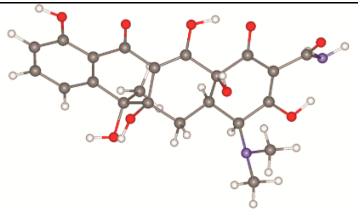<br>Nastya-4<br>**C9**<br>E= -1637,853097; G=236.3;<br>ΔE=5.4; ΔG=5.7;	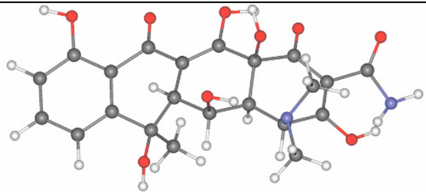<br>Nastya-6<br>**C15**<br>E= -1637,852767; G=237.0;<br>ΔE=5.6; ΔG=6.6;	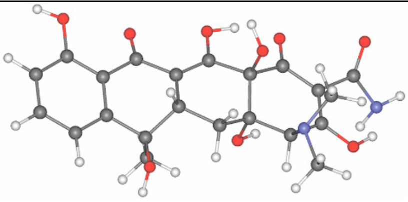<br>Nastya-7<br>**C14**<br>E= -1637,849442; G=235.6;<br>ΔE=7.7; ΔG=7.3
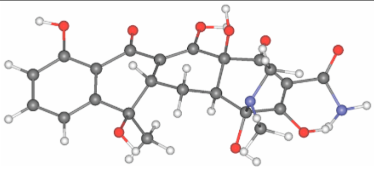<br>Nastya-8<br>**C19**<br>E= -1637,851738; G=236.0;<br>ΔE=6.3; ΔG=6.3;		
